# Genomic Characterisation of the Indigenous Irish Kerry Cattle Breed

**DOI:** 10.3389/fgene.2018.00051

**Published:** 2018-02-19

**Authors:** Sam Browett, Gillian McHugo, Ian W. Richardson, David A. Magee, Stephen D. E. Park, Alan G. Fahey, John F. Kearney, Carolina N. Correia, Imtiaz A. S. Randhawa, David E. MacHugh

**Affiliations:** ^1^Ecosystems and Environment Research Centre, School of Environment and Life Sciences, University of Salford, Salford, United Kingdom; ^2^Animal Genomics Laboratory, UCD School of Agriculture and Food Science, University College Dublin, Dublin, Ireland; ^3^IdentiGEN Ltd., Dublin, Ireland; ^4^Irish Cattle Breeding Federation, Bandon, Ireland; ^5^Sydney School of Veterinary Science, The University of Sydney, Camden, NSW, Australia; ^6^UCD Conway Institute of Biomolecular and Biomedical Research, University College Dublin, Dublin, Ireland

**Keywords:** cattle, conservation genomics, endangered breed, inbreeding, genetic diversity, population genomics, selection signature, single nucleotide polymorphism

## Abstract

Kerry cattle are an endangered landrace heritage breed of cultural importance to Ireland. In the present study we have used genome-wide SNP array data to evaluate genomic diversity within the Kerry population and between Kerry cattle and other European breeds. Patterns of genetic differentiation and gene flow among breeds using phylogenetic trees with ancestry graphs highlighted historical gene flow from the British Shorthorn breed into the ancestral population of modern Kerry cattle. Principal component analysis (PCA) and genetic clustering emphasised the genetic distinctiveness of Kerry cattle relative to comparator British and European cattle breeds. Modelling of genetic effective population size (*N*_e_) revealed a demographic trend of diminishing *N*_e_ over time and that recent estimated *N*_e_ values for the Kerry breed may be less than the threshold for sustainable genetic conservation. In addition, analysis of genome-wide autozygosity (*F*_ROH_) showed that genomic inbreeding has increased significantly during the 20 years between 1992 and 2012. Finally, signatures of selection revealed genomic regions subject to natural and artificial selection as Kerry cattle adapted to the climate, physical geography and agro-ecology of southwest Ireland.

## Introduction

Approximately 10,000 years ago, humans first domesticated wild aurochs (*Bos primigenius*)—the progenitor of modern cattle—in the Fertile Crescent region of Southwest Asia (Larson and Fuller, 2014; Larson et al., 2014; MacHugh et al., 2017). Extant domestic cattle, which encompass humpless taurine (*B. taurus*), humped zebu (*B. indicus*) and myriad *B. taurus/indicus* hybrid populations, have, through genetic drift and natural and artificial selection, diversified into more than 1,100 recognised breeds. However, beginning in the middle of the twentieth century, socioeconomic preferences for large highly productive dairy, beef and dual-purpose breeds have led to extinction and increased vulnerability of more than 200 locally-adapted landrace or native cattle breeds (Gandini et al., 2004; Food and Agriculture Organization, 2007, 2015).

With the advent of accelerating climate change, particularly in the Arctic and circumarctic regions (Vihma, 2014; Gao et al., 2015), agro-ecological environments in north-western Europe will inevitably undergo significant change during the coming century (Smith and Gregory, 2013; Wheeler and von Braun, 2013). It is, therefore, increasingly recognised that long-term sustainability of animal production systems and food security will necessitate conservation and management of livestock genetic resources in this region (Hoffmann, 2010; Boettcher et al., 2015; Kantanen et al., 2015). Locally-adapted native livestock breeds with distinct microevolutionary histories and minimal external gene flow will have accumulated novel genomic variation and haplotype combinations for quantitative health, fertility and production traits (Hill, 2014; Felius et al., 2015; Kristensen et al., 2015). These populations may therefore be key to future breeding programmes directed towards adaptation of European livestock to new agro-ecological and production environments (Biscarini et al., 2015; Boettcher et al., 2015; Phocas et al., 2016a,b).

The availability of powerful and cheap tools for genotyping large numbers of single nucleotide polymorphisms (SNPs) has provided conservation biologists and animal geneticists with the opportunity to characterise genomic variation and estimate population genetic parameters at very high resolution in threatened or endangered livestock breeds (Pertoldi et al., 2014; Ben Jemaa et al., 2015; Beynon et al., 2015; Mészáros et al., 2015; Burren et al., 2016; Decker et al., 2016; Iso-Touru et al., 2016; Manunza et al., 2016; Mastrangelo et al., 2016; Visser et al., 2016; Williams et al., 2016; François et al., 2017). These studies are already providing important baseline data for genetic conservation and will underpin programmes for managed breeding and biobanking of these populations (Groeneveld et al., 2016).

As a native breed with a claimed ancient heritage, Kerry cattle are considered culturally important to Ireland (Curran, 1990). It is a landrace cattle population that remains productive in harsh upland regions with poor quality feed, which are typical of southwest Ireland where the Kerry breed evolved (Food and Agriculture Organization, 2017). These cattle were often referred to anecdotally in Ireland as the “poor man's cow” due to their ability to produce relatively large quantities of milk on very sparse fodder; the Kerry breed is also considered to be a remnant of what was once a substantially larger and more widespread historical population. Levels of inbreeding have been estimated using pedigree data and the accumulated figure since the foundation of the herd book in 1887 reached 15% in 1985 (O'hUigín and Cunningham, 1990).

In recent decades the Kerry cattle breed has experienced significant population fluctuations due to changing socioeconomic and agricultural circumstances. During the 1980s, the number of breeding females decreased to less than 200, prompting the Irish agricultural authorities to introduce a Kerry cattle conservation scheme (McParland, 2013), which has continued to the present day in the form of the Department of Agriculture, Food and the Marine (DAFM) Kerry Cattle Premium Scheme (Department of Agriculture Food and the Marine, 2017).

The formal conservation policy and supports initiated during the early 1990s led to a significant increase in the Kerry cattle population, such that by 2007 the number of breeding females had increased to more than a thousand animals (Food and Agriculture Organization, 2007). In recent years, however, due to deteriorating economic circumstances in Ireland post-2008, the Kerry cattle population has substantially declined once again and is classified as endangered and under significant threat of extinction or absorption through crossbreeding with other breeds (McParland, 2013; Department of Agriculture Food and the Marine, 2014).

The Kerry cattle breed was one of the first European heritage cattle breeds to be surveyed using molecular population genetics techniques. We have previously used autosomal microsatellite genetic markers and mitochondrial DNA (mtDNA) control region sequence variation for comparative evolutionary studies of genetic diversity in Kerry cattle and other British, European, African and Asian breeds (MacHugh et al., 1997, 1998, 1999; Troy et al., 2001). In addition, Bray et al. have used microsatellites to examine admixture and ancestry in Kerry cattle and the Dexter and Devon breeds (Bray et al., 2009). Results from these studies demonstrated that Kerry cattle exhibit markedly low mtDNA sequence diversity, but autosomal microsatellite diversity comparable to other cattle breeds native to Britain and Ireland. More recently, analyses of medium- and high-density SNP genotypes generated using genome sequence data from an extinct British *B. primigenius* subfossil have shown that Kerry cattle retain a significant genomic signature of admixture from wild aurochs (Park et al., 2015; Upadhyay et al., 2017). This observation highlights the genetic distinctiveness of the Kerry population and has major implications for conservation and management of the breed.

For the present study, and within a genetic conservation framework, we performed high-resolution comparative population genomics analyses of Kerry cattle and a range of British and European cattle breeds. These analyses encompassed phylogenetic network reconstruction, evaluation of genetic structure and inbreeding, modelling of historical effective population sizes and functional analyses of artificial and natural selection across the Kerry genome.

## Materials and methods

### Kerry cattle population DNA sampling in 1991/92 and 2011/12

**Figure 1:**
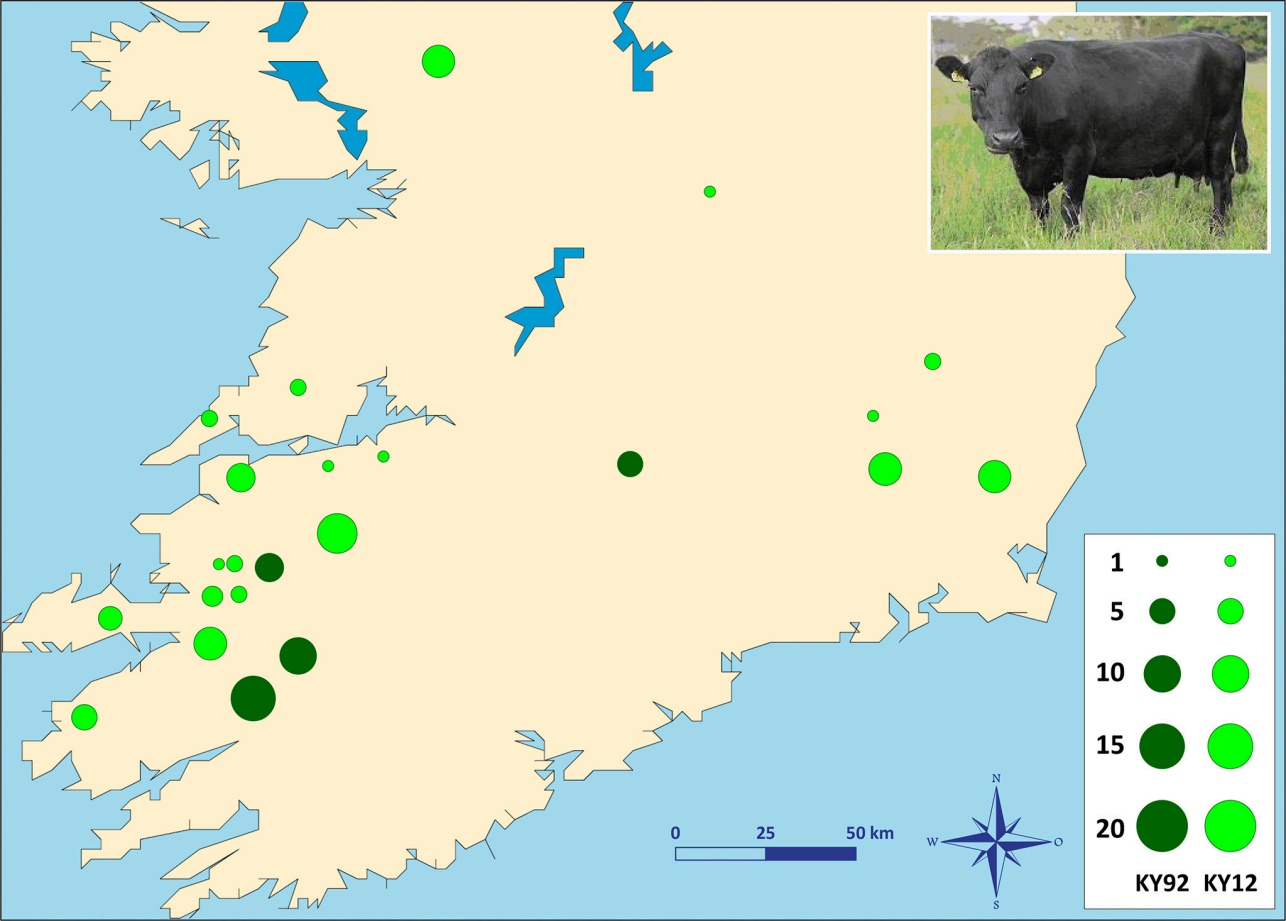
Photograph of a Kerry cow and locations of Kerry cattle herd DNA sampling in southern Ireland. The area of each circle corresponds to the size of each population sample. Dark green = animals sampled during 1991/92 (KY92); light green = animals sampled during 2011/12 (KY12). Kerry cow image is copyright of the Kerry Cattle Society Ltd.

Two different population samples from the Irish Kerry cattle breed were used for this study (Figure 1). The first population sample consisted of peripheral blood and semen straw genomic DNA collected and purified from 36 male and female Kerry cattle in 1991/92, which are a subset of the Kerry cattle population sample (*n* = 40) we have previously described and used for microsatellite-based population genetics analyses (MacHugh et al., 1997, 1998). Pedigree records and owners were consulted to ensure that a representative sample of animals was obtained. This Kerry population sample group is coded as KY92.

The second Kerry cattle population sample was collected in 2011/12 from 19 different herds located across southern and western Ireland. Performagene (PG-100) nasal swab DNA collection kits were used for biological sample collection (DNA Genotek Inc., Ottawa, Canada). Nasal swab DNA samples were collected from a total of 75 male and female Kerry cattle and owners were consulted to ensure that a representative sample of animals was obtained. This Kerry population sample group is coded as KY12. Genomic DNA was purified from 0.5 ml of each PG-100 nasal swab sample using the laboratory protocol recommended by the manufacturer (DNA Genotek Inc.).

### SNP genotyping and assembly of comparative SNP data sets

Illumina® Bovine SNP50 BeadChip (Matukumalli et al., 2009) genotyping on all 111 Kerry genomic DNA samples (KY92 and KY12 sample panels plus nine blinded sample duplicates for quality control purposes) was performed by Weatherbys Scientific (Co. Kildare, Ireland).

For comparative population genomics analyses, equivalent SNP data for a range of other breeds were obtained from previously published work (Decker et al., 2009; Flori et al., 2009; Gibbs et al., 2009; Matukumalli et al., 2009; Gautier et al., 2010; Park et al., 2015). The breed SNP data were split into two discrete composite data sets: a European breed SNP data set (EU) and a SNP data set for a subset of European breeds originating from Britain and Ireland (BI). A population sample of West African N'Dama *B. taurus* cattle from Guinea (NDAM) was also used as an outgroup for the phylogenetic analyses. Table 1 provides detailed biogeographical information on the cattle breed samples used for the present study.

**Table 1:** Cattle breed/population samples used for the present study.

**Breed/population**	**Code**	**Data set**	**Breed purpose**	**Country of origin**	**Source**
Angus	ANGU	BI/EU	Beef	Scotland	1, 2
Belted Galloway	BGAL	BI	Beef	Scotland	2
British Shorthorn	BSHN	BI/EU	Dual purpose	England	2
Brown Swiss	BRSW	EU	Dairy	Switzerland	1, 2, 3
Charolais	CHAR	EU	Beef	France	1, 2, 3
Devon	DEVN	BI	Beef	England	2
Dexter	DXTR	BI	Dual purpose	Ireland	2
English Longhorn	ELHN	BI	Beef	England	2
Finnish Ayrshire	FAYR	BI/EU	Dairy	Scotland/Finland	2
Galloway	GALL	BI	Beef	Scotland	2
Gelbvieh	GELB	EU	Dual purpose	Germany	2, 4
Guernsey	GNSY	BI/EU	Dairy	Channel Islands	1, 2
Hereford	HRFD	BI/EU	Beef	England	1, 2
Holstein	HOLS	EU	Dairy	The Netherlands	1, 2, 5
Jersey	JRSY	BI/EU	Dairy	Channel Islands	1, 2, 3
Kerry sampled 1991/92	KY92	BI/EU	Dairy	Ireland	Current
Kerry sampled 2011/12	KY12	BI/EU	Dairy	Ireland	Current
Limousin	LIMS	EU	Beef/draft	France	1, 2
Lincoln Red	LNCR	BI	Beef	England	2
Montbeliarde	MONT	EU	Dairy	France	2, 5
N'Dama	NDAM	–	Dual purpose	Guinea (West Africa)	1
Norwegian Red	NRED	EU	Dairy	Norway	1
Piedmontese	PDMT	EU	Dual purpose	Italy	1, 2
Red Angus	RANG	BI/EU	Beef	Scotland	1
Red Poll	REDP	BI	Beef	England	2
Romagnola	ROMG	EU	Beef/draft	Italy	1
Scottish Highland	SCHL	BI	Beef	Scotland	2
Simmental	SIMM	EU	Dual purpose/draft	Switzerland	2, 4
South Devon	SDEV	BI	Beef	England	2
Sussex	SUSX	BI	Beef/draft	England	2
Welsh Black	WBLK	BI	Dual purpose	Wales	2
White Park	WHPK	BI	Dual purpose/draft	England	2

*^1^Gibbs et al. (2009); ^2^Decker et al. (2009); ^3^Gautier et al. (2010); ^4^Matukumalli et al. (2009); ^5^Flori et al. (2009)*.

### Sample removal and quality control and filtering of SNPs

Genomic non-exclusion—in other words, genome-wide SNP profiles completely compatible with parent-offspring relationship (with allowance for very low-level genotyping error)—were used to identify animals from the KY92 and KY12 population samples that were parent-offspring pairs. One of the two animals in each pair was then randomly removed to generate the working SNP data set. Following this procedure, quality control and filtering based on recorded SNP genotypes was performed as detailed below for the EU and BI data sets.

Prior to quality control and filtering there were 54,057 SNPs in the EU data set (608 animals, including KY92 and KY12) and in the BI data set (354 animals, including KY92 and KY12). SNP quality filtering was performed using PLINK version 1.07 (Purcell et al., 2007), such that individual SNPs with more than 10% missing data and a minor allele frequency (MAF) of ≤0.01 (1%) were removed from both data sets; however, for analyses of genomic inbreeding and runs of homozygosity (ROH) the MAF filtering threshold was not imposed. Only autosomal SNPs were retained and individual animal samples with a SNP call rate less than 90% were also removed from each of the two data sets.

SNP quality control and filtering were performed across breeds/populations (by data set) for construction of phylogenies and ancestor graphs, multivariate analysis, investigation of population structure and detection of signatures of selection. For intrapopulation analyses of effective population size (*N*_e_) and genomic inbreeding, all SNPs genotyped (54,057) were filtered within breeds/populations as detailed above. However, an additional filtering procedure was used to remove SNPs deviating from Hardy-Weinberg equilibrium (HWE) with a *P*-value threshold of < 0.0001. Also, for the *N*_e_ analysis, a more stringent MAF threshold of 0.05 was used.

### Generation of identity-by-state (IBS) matrix

Using the filtered genome-wide SNP data, PLINK v1.07 was also used to generate identity-by-state (IBS) values for all pairs of Kerry cattle (KY92 and KY12), including the nine blinded sample duplicates for sample verification and tracking purposes.

### Construction of phylogenetic trees and ancestry graphs

Maximum likelihood (ML) phylogenetic trees with ancestry graphs were generated for the EU and BI data sets using the TreeMix (version 1.12) software package (Pickrell and Pritchard, 2012). The West African *B. taurus* NDAM breed sample (*n* = 22) was used as an outgroup. TreeMix was run without using SNP blocks (as described in the TreeMix software documentation) and ML phylogenetic trees were generated with no migration edges (*m* = 0) up to ten migration edges (*m* = 10).

### Population differentiation and genetic structure

To visualise the main axes of genomic variation among cattle breeds and individual animals, multivariate principal component analysis (PCA) was performed for the composite EU and BI SNP data sets using SMARTPCA from the EIGENSOFT package (version 4.2) with default settings (Patterson et al., 2006).

To further investigate genetic structure and admixture history for Kerry cattle and other breeds the fastSTRUCTURE software package (Raj et al., 2014) was used to analyse the EU and BI data sets for a range of *K* possible ancestral populations (*K* = 2–15). For the present study, the simple prior approach described by Raj et al. (2014) was used, which is sufficient for modelling population/breed divergence. To identify the “true” *K*-value for the number of ancestral populations, a series of fastSTRUCTURE runs with pre-defined *K*-values were examined using the *chooseK.py* script (Raj et al., 2014). Outputs from the fastSTRUCTURE analyses were visualised using the DISTRUCT software program (Rosenberg et al., 2002) using standard parameters.

### Modelling current and historical effective population size (*N*_e_)

Current and historical *N*_e_ trends were modelled with genome-wide SNP linkage disequilibrium (LD) data for the KY92 and KY12 populations plus a selection of BI and EU breeds using the SNeP software tool as described by Barbato et al. (2015). This method facilitates estimation of historical *N*_e_ values from SNP linkage disequilibrium (LD) data using the following equation (Corbin et al., 2012):

$$N_{T(t)}={\left(4f\left(c_{t}\right)\right)}^{-1}\left(E{\left[r_{adj}^{2}|c_{t}\right]}^{-1}-\alpha \right)$$

where *N*_*T*_ is the effective population size *t* generations ago calculated as $t={\left(2f\left(c_{t}\right)\right)}^{-1}$ (Hayes et al., 2003), *c*_*t*_ is the recombination rate defined for a specific physical distance between SNP markers, $r_{adj}^{2}$ is the LD value adjusted for sample size and the recommended default α value = 1 was used to correct for the occurrence of mutation (Barbato et al., 2015). In addition, the SNeP program option for unphased SNP data was used for the analyses described here.

### Evaluation of genomic inbreeding and runs of homozygosity (ROH)

Individual animal genomic inbreeding was evaluated as genome-wide autozygosity estimated from SNP data using ROH and the *F*_ROH_ statistic introduced by McQuillan et al. (2008). The *F*_ROH_ statistic was calculated as the ratio of the total length of defined ROH (*L*_ROH_) to the total length of the autosomal genome covered by SNPs:

$$F_{ROH}=\frac{\sum L_{ROH}}{L_{AUTO}}$$

PLINK v1.07 was used to define ROH using a sliding window approach and procedures modified from previous recommendations for Illumina® Bovine SNP50 BeadChip and similar SNP data sets (Purfield et al., 2012, 2017). The criteria for defining individual ROH were set such that the ROH was required to be at least 500 kb in length, with a minimum density of one SNP per 120 kb and that there was a gap of at least 1,000 kb between each ROH. A sliding window of 50 SNPs was incrementally advanced one SNP at a time along the genome; each discrete window could contain a maximum of one heterozygous SNP and no more than two SNPs with missing genotypes. Following Purfield et al. (2012) all filtered genomic SNPs (without a MAF threshold), including those located in centromeric regions, were used to estimate *F*_ROH_ values for individual animals.

### Genome-wide detection of signatures of selection and functional enrichment analysis

In the absence of hard selective sweeps, single selection tests using high-density SNP data do not perform well in detecting signatures of selection from individual livestock breeds (Kemper et al., 2014). Therefore, for the present study, genomic signatures of selection were identified using the composite selection signal (CSS) method introduced by Randhawa et al. (2014). The CSS method has been shown to be a robust and sensitive approach for detecting genomic signatures of selection underlying microevolution of complex traits in livestock (Randhawa et al., 2015). The CSS is a weighted index of signatures of selection from multiple estimates; it is a nonparametric procedure that uses fractional ranks of constituent tests and does not depend on assumptions about the distributions of individual test results.

As described in detail by Randhawa et al. (2014), the CSS method can be used to combine the fixation index (*F*_ST_), the change in selected allele frequency (Δ*SAF*) and the cross-population extended haplotype homozygosity (*XP-EHH*) tests into one composite statistic for each SNP in a population genomics data set. For the present study, we used 36,621 genome-wide SNPs genotyped in 98 individual Kerry cattle samples (from both the KY92 and KY12 populations) and a sample of 102 randomly selected cattle (six random cattle from each breed of the EU data set). To mitigate against false positives, genomic selection signatures were only considered significant if at least one SNP from the set of the top 0.1% genome-wide CSS scores was flanked by at least five SNPs from the set of the top 1% CSS scores.

The Ensembl BioMart data mining resource (Smedley et al., 2015) was used to identify genes within ± 1.0 Mb of each selection peak (Ensembl release 90, August 2017). Following this, Ingenuity® Pathway Analysis (IPA®: Qiagen, Redwood City, CA, USA; release date June 2017) was used to perform an overrepresentation enrichment analysis (ORA) with this gene set to identify canonical pathways and functional processes of biological importance. The total gene content of Ensembl release 90 version of the UMD3.1 bovine genome assembly (Zimin et al., 2009) was used as the most appropriate reference gene set for these analyses (Timmons et al., 2015).

## Results and discussion

### Sample removal and SNP filtering and quality control

Genomic non-exclusion identified 20 parent-offspring pairs from the KY12 population and one sample from each pair was randomly removed (sample codes: KY12_01, KY12_05, KY12_13, KY12_14, KY12_17, KY12_18, KY12_19, KY12_46, KY12_55, KY12_67). Thereafter, general SNP quality control and filtering led to additional samples being excluded (KY12_26, KY12_28 and KY12_54), giving a total filtered KY12 population sample of 62 animals for downstream population genomics analyses.

After SNP quality control and filtering across the two composite data sets (EU and BI), there were 36,621 autosomal SNPs from 605 individual animals in the EU data set and there were 37,395 autosomal SNPs from 351 animals in the BI data set. When the West African NDAM breed sample (*n* = 22) was included for the ML phylogenetic tree and ancestry graph analyses, the number of SNPs used was 36,000 from 627 animals for the EU data set and 37,490 from 373 animals for the BI data set. The final numbers of SNPs used for individual breed/population analyses of *N*_e_ and genomic inbreeding after all quality control and filtering (including additional filtering for deviations from HWE) are shown in Table 2.

**Table 2:** Breed/population sample size, observed heterozygosity and SNP filtering information.

**Breed/population**	**Code**	**Data set**	**Sample size (*n*) post-filtering**	***Observed heterozygosity H*_o_**	**No. SNPs *N*_e_ modelling**	**No. SNPs genomic inbreeding**
Angus	ANGU	BI/EU	72	0.3048	31,413	39,576
Belted Galloway	BGAL	BI	4	0.2902	25,997	39,582
British Shorthorn	BSHN	BI/EU	10	0.2549	29,038	39,576
Brown Swiss	BRSW	EU	31	0.2894	–	–
Charolais	CHAR	EU	48	0.3209	–	–
Devon	DEVN	BI	4	0.2859	–	–
Dexter	DXTR	BI	4	0.2458	24,753	37,903
English Longhorn	ELHN	BI	3	0.2232	–	–
Finnish Ayrshire	FAYR	BI/EU	7	0.3064	–	–
Galloway	GALL	BI	4	0.2942	–	–
Gelbvieh	GELB	EU	8	0.3125	–	–
Guernsey	GNSY	BI/EU	19	0.2764	–	50,323
Hereford	HRFD	BI/EU	35	0.2964	–	39,535
Holstein	HOLS	EU	70	0.3192	36,152	43,135
Jersey	JRSY	BI/EU	44	0.2718	31,358	43,136
Kerry sampled 1991/92	KY92	BI/EU	36	0.2965	37,556	51,731
Kerry sampled 2011/12	KY12	BI/EU	62	0.3042	36,428	50,756
Limousin	LIMS	EU	45	0.3122	–	–
Lincoln Red	LNCR	BI	7	0.2789	26,350	34,173
Montbeliarde	MONT	EU	31	0.3019	–	–
N'Dama	NDAM	–	22	0.2158	–	–
Norwegian Red	NRED	EU	20	0.3190	–	–
Piedmontese	PDMT	EU	23	0.3240	–	–
Red Angus	RANG	BI/EU	14	0.3092	–	–
Red Poll	REDP	BI	5	0.2905	–	–
Romagnola	ROMG	EU	21	0.2943	–	–
Scottish Highland	SCHL	BI	8	0.2823	–	–
Simmental	SIMM	EU	9	0.3136	–	–
South Devon	SDEV	BI	3	0.3070	–	–
Sussex	SUSX	BI	4	0.2792	–	–
Welsh Black	WBLK	BI	2	0.3203	–	–
White Park	WHPK	BI	4	0.2270	–	–

All data sets, including EU and BI composite data sets and individual breed/population data sets had total SNP call rates of > 99%. The IBS values estimated for Kerry cattle (KY92 and KY12) from filtered genome-wide SNP data are reported in Supplementary Table 1 and described further in section Genomic Relationship and Analysis of Inbreeding.

### Observed heterozygosity (*H*_o_) estimated from genome-wide SNP data

Table 2 provides genome-wide *H*_o_ values for each of the breeds/populations used for the present study. The lowest genome-wide *H*_o_ value was observed for the West Africa NDAM *B. taurus* breed, which is likely a consequence of ascertainment bias introduced by a focus on polymorphic SNPs in European *B. taurus* during design of the Illumina® Bovine SNP50 BeadChip (Matukumalli et al., 2009).

Generally, as shown in Table 2 for the EU and BI breeds and populations, local landrace or heritage breeds display lower *H*_o_ values compared to more widespread production breeds such as the Simmental (SIMM), Holstein (HOLS) or Charolais (CHAR) breeds. In addition, as might be expected, production breeds originally derived from minor island populations (Jersey [JRSY] and Guernsey [GNSY]) also exhibit relatively low *H*_o_ values. In the context of genetic conservation it is therefore encouraging that the KY92 and KY12 population samples display intermediate *H*_o_ values that are at the upper end of the range observed for the heritage breeds.

### Maximum likelihood phylogenetic ancestry graphs using genome-wide SNP data

**Figure 2:**
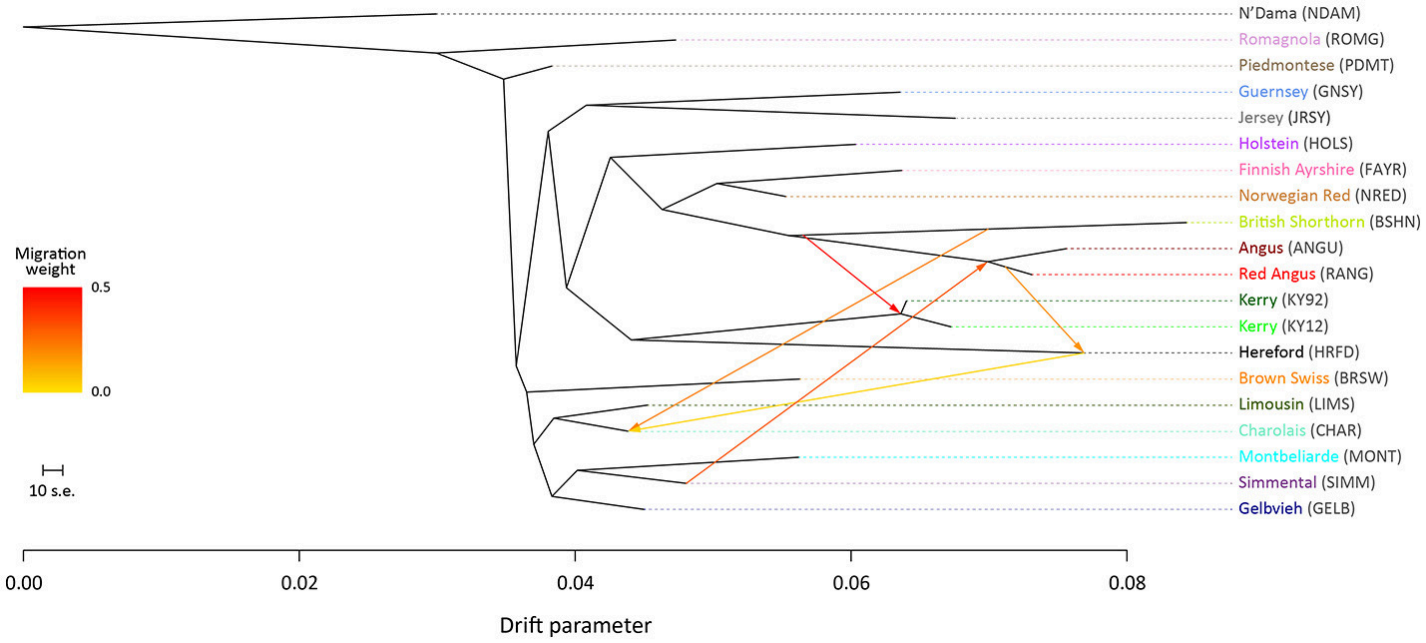
Maximum likelihood (ML) phylogenetic tree network graph with five migration edges (*m* = 5) generated for genome-wide SNP data (36,000 autosomal SNPs) from European cattle breeds (EU data set). The West African taurine N'Dama breed sampled in Guinea is included as a population outgroup. Coloured lines and arrows show migration edges that model gene flow between lineages with different migration weights represented by the colour gradient.

To examine microevolutionary patterns of genetic differentiation and gene flow among cattle breeds and populations, ML phylogenetic ancestry graphs were generated using TreeMix. For the EU data set, the ML tree topology was consistent for all values of *m*, with the exception of *m* = 2 migration edges, where the Hereford breed (HRFD) was observed to group with the HOLS breed. The ML tree generated with *m* = 5 is shown in Figure 2, which highlights the genetic similarity of the Northern European breeds (British, Irish and Scandinavian). As expected the two Kerry population samples (KY92 and KY12) are genetically very similar and emerge on the same branch as the HRFD breed. It is also noteworthy that there is a high-weight migration edge between the British Shorthorn breed (BSHN) and the root of the two Kerry population samples, supporting the hypothesis of historical gene flow from the British Shorthorn breed into the ancestral population of modern Kerry cattle (Curran, 1990).

**Figure 3:**
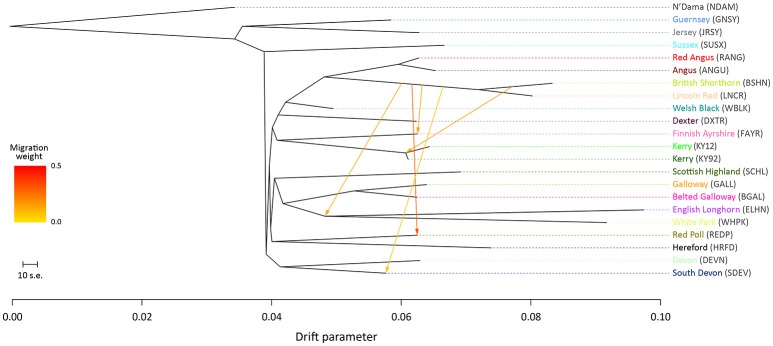
Maximum likelihood (ML) phylogenetic tree network graph with five migration edges (*m* = 5) generated for genome-wide SNP data (37,490 autosomal SNPs) from cattle breeds of British and Irish origin (BI data set). The West African taurine N'Dama breed sampled in Guinea is included as a population outgroup. Coloured lines and arrows show migration edges that model gene flow between lineages with different migration weights represented by the colour gradient.

For the ML trees generated using the BI data set, breed/population differentiation was less apparent, possibly due to similar biogeographical origins for these breeds and/or smaller sample sizes for some of the populations sampled. Figure 3 shows the ML tree generated with *m* = 5 for the BI data set. For *m* = 5, all migration edges stem from the BSHN/Lincoln Red (LNCR) branch, including a medium-weight migration edge connecting to the Kerry cattle branch. These results support the hypothesis that there was significant gene flow during the eighteenth and nineteenth centuries from British Shorthorn cattle into the ancestral populations for a range of modern British and Irish cattle breeds (Grobet et al., 1998; Felius et al., 2011, 2015).

### Multivariate principal component analysis of genome-wide SNP data

**Figure 4:**
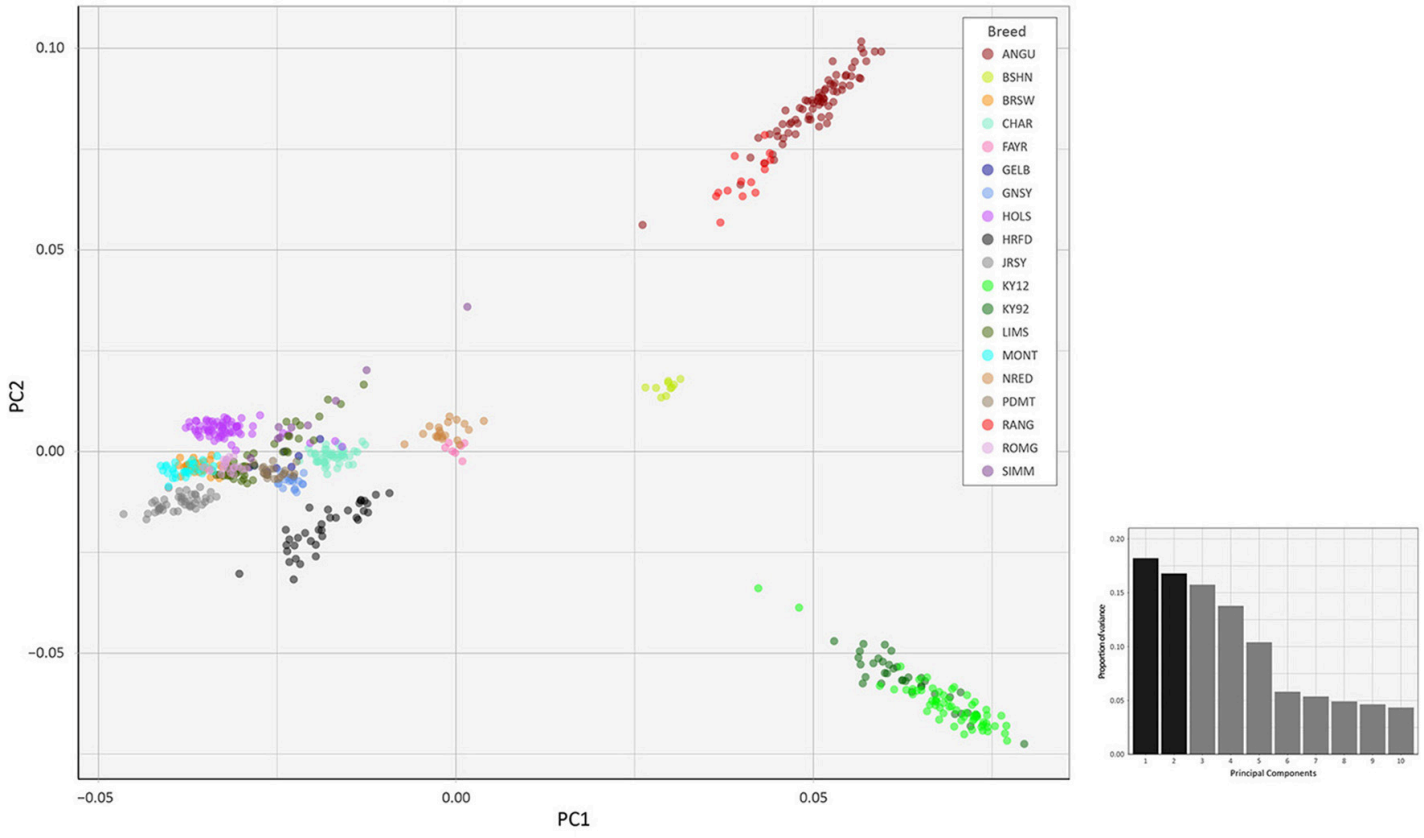
Principal component analysis plot constructed for PC1 and PC2 from genome-wide SNP data (36,621 autosomal SNPs) for the EU data set of 605 individual animals. The smaller histogram plot shows the relative variance contributions for the first 10 PCs and PC1 and PC2 account for 18.2% and 16.8% of the total variation for PC1–10, respectively.

**Figure 5:**
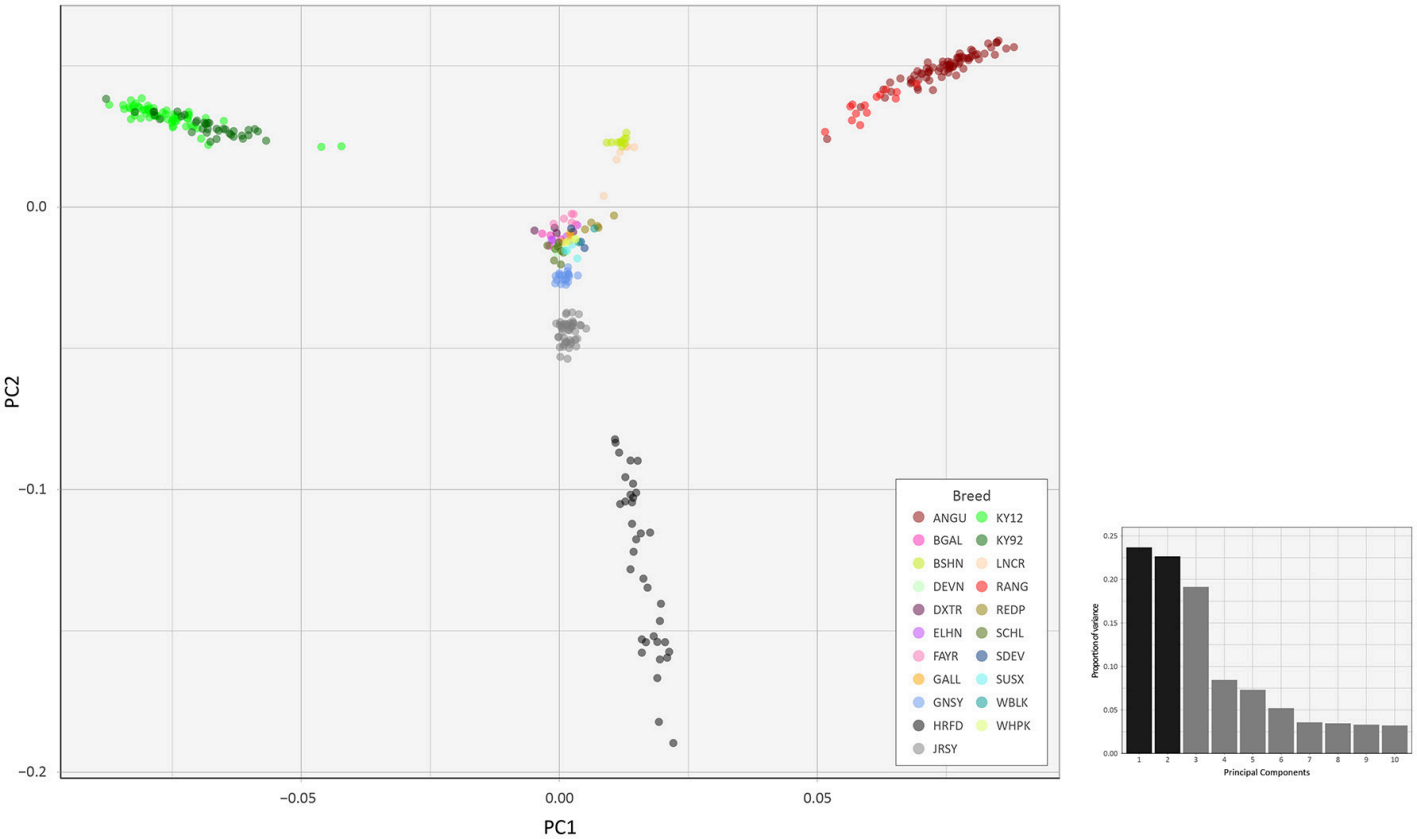
Principal component analysis plot constructed for PC1 and PC2 from genome-wide SNP data (37,395 autosomal SNPs) for the BI data set of 351 individual animals. The smaller histogram plot shows the relative variance contributions for the first 10 PCs and PC1 and PC2 account for 23.7% and 22.7% of the total variation for PC1–10, respectively.

To investigate inter- and intra-population genomic diversity and genetic relationship among individual animals from multiple cattle breeds and populations, PCA was performed using genome-wide SNP data. Principal component plots of the first (PC1) and second (PC2) principal components are shown in Figures 4, 5 for the EU and BI data sets, respectively.

In Figure 4, for the EU data set, PC1 and PC2 account for 18.2% and 16.8% of the total variation for PC1–10, respectively. The PC1 plot axis differentiates the British Angus (ANGU), Red Angus (RANG) and BSHN and Irish KY92 and KY12 populations from the rest of the European breeds, including the British HRFD and GNSY and JRSY Channel Islands breeds. In addition, the ANGU and RANG and the Kerry (KY92 and KY12) emerge at the opposite extremes of the PC2 plot axis. In Figure 5, for the BI data set, PC1 and PC2 account for 23.7% and 22.7% of the total variation for PC1–10, respectively. The PC1 plot axis recapitulates PC2 in Figure 4 and differentiates the Kerry (KY92 and KY12) from the ANGU and RANG breeds with the other British breeds emerging between these two extremes. These results highlight the genetic distinctiveness of the Kerry cattle breed in comparison to a wide range of British production and heritage landrace cattle breeds and support their status as an important cattle genetic resource that should be prioritised for conservation.

The PC2 plot axis in Figure 5 differentiates the HRFD breed from the other British and Irish breeds and reveals substantial genetic diversity among individual HRFD animals. However, in this context, it is important to note that the pattern of genetic diversity revealed here for the HRFD population sample may be due to ascertainment bias as a consequence of the strategy used to design the Illumina® Bovine SNP50 BeadChip. In this regard, many of the SNPs that constitute this first-generation SNP array were identified from heterozygous positions in the inbred Hereford female (L1 Dominette 01449) bovine genome assembly or through comparisons of random shotgun reads from six diverse cattle breeds that were aligned directly to the same Hereford genome assembly (Matukumalli et al., 2009). This approach to SNP array design will inevitably lead to elevated intrabreed genomic variation using the Illumina® Bovine SNP50 BeadChip with Hereford cattle (Meuwissen, 2009) and accounts for the dispersed pattern of individual HRFD samples in Figure 5.

Examination of Figures 4, 5 indicates that two of the KY12 animals sampled may exhibit a genetic signature of ancestral crossbreeding with another cattle population, which, anecdotally, is likely to have been due to crossbreeding with Angus cattle. Therefore, another PCA plot was generated (Supplementary Figure 1) that shows PC1 and PC2 for individual animals from the KY92, KY12, ANGU and RANG population samples. The two animals exhibiting a genetic signature of possible crossbreeding (KY12_06 and KY12_58) are indicated on Supplementary Figure 1. Notwithstanding the KY12_06 and KY12_58 data points, the genetic similarity among all Kerry cattle sampled is evident by comparison of the tight KY92 and KY12 sample cluster to the dispersion of the ANGU and RANG samples on the PCA plot in Supplementary Figure 1.

The values for the variation accounted for by PC3, PC4 and PC5 in Figure 4 (EU data set) are relatively high (15.7, 13.8, and 10.4%, respectively). For Figure 5 (BI data set), the variation accounted for by PC3 is also relatively high (19.1%). Therefore, we generated additional PCA plots of PC1 for each of the two data sets vs. these additional principal components (see Supplementary Figures 2–5).

### Analysis of genetic structure using genome-wide SNP data

**Figure 6:**
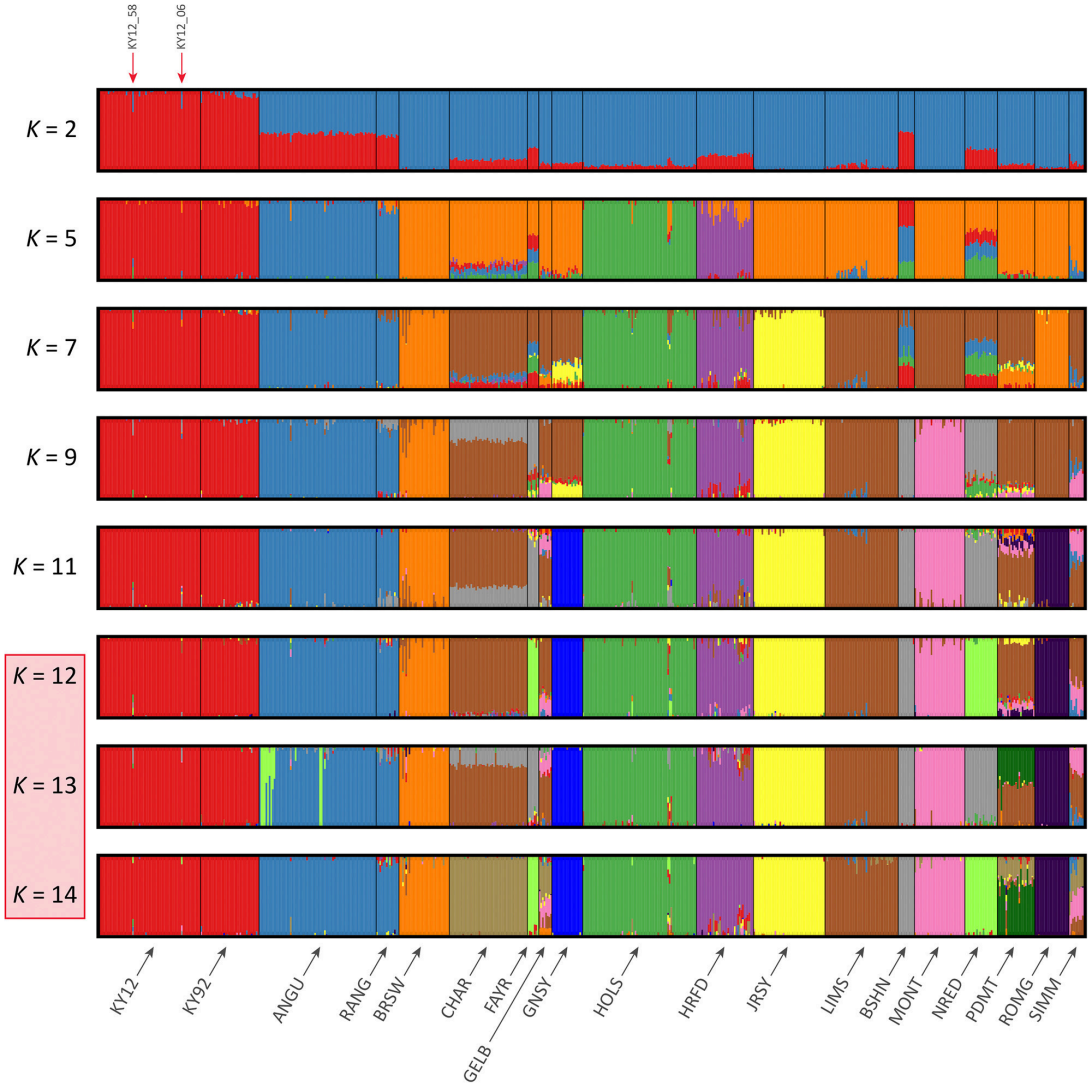
Hierarchical clustering of individual animals using genome-wide SNP data (36,621 autosomal SNPs) for the EU data set of 605 individual animals. Results are shown for modelled ancestral populations *K* = 2–14. The cluster numbers corresponding to the likely number of ancestral populations are highlighted with a light red overlay and the two outlier Kerry samples (KY12_06 and KY12_58) are indicated with red arrows.

**Figure 7:**
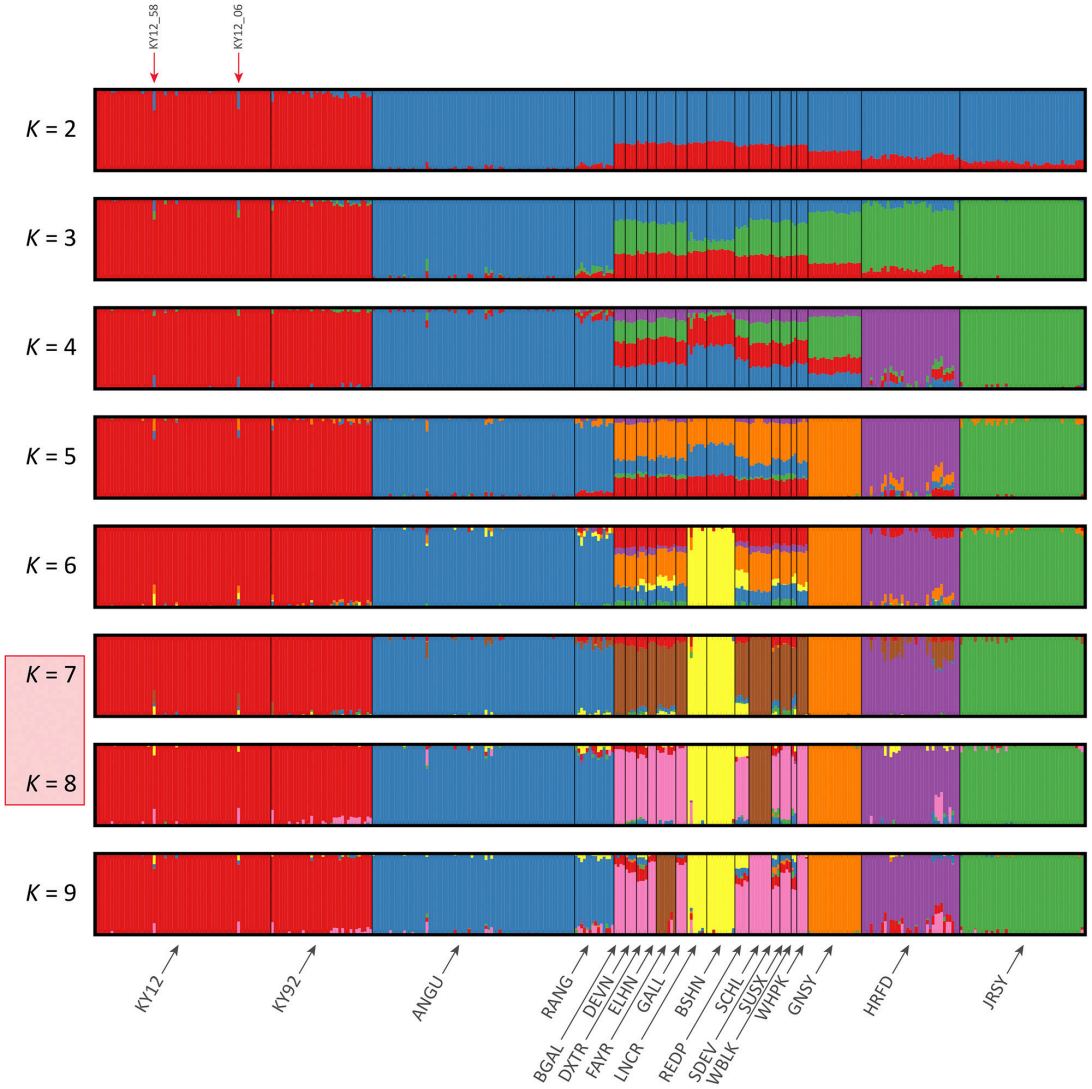
Hierarchical clustering of individual animals using genome-wide SNP data (37,395 autosomal SNPs) for the BI data set of 351 individual animals. Results are shown for modelled ancestral populations *K* = 2–9. The cluster numbers corresponding to the likely number of ancestral populations are highlighted with a light red overlay and the two outlier Kerry samples (KY12_06 and KY12_58) are indicated with red arrows.

The results of the fastSTRUCTURE analyses using the EU and BI data sets are shown in Figures 6, 7, respectively. For both analyses, the Kerry cattle (KY92 and KY12) cluster as a single group at *K* = 2 and are differentiated from all other European or British and Irish cattle breeds. The other breed group that is clearly differentiated at *K* = 2 in Figure 7 is the cluster composed of the ANGU and RANG breeds. These results mirror the pattern shown for PC1 in Figure 5, and again emphasise the genetic distinctiveness of Kerry cattle compared to other European production and landrace heritage breeds. Using the *chooseK.py* script the “true” number of clusters corresponding to the likely number of ancestral populations was estimated to be between 12 and 14 for the EU data set and either 7 or 8 for the BI data set.

For both data sets, animals from the KY12 population sample appear to be more genetically homogenous compared to the KY92 population sample. This observation may be a consequence of increasing use, since the early 1990s, of small numbers of artificial insemination (AI) Kerry sires. It is also noteworthy that the two individual animals detected with a substantial signature of putative historical crossbreeding (KY12_06 and KY12_58) show marked patterns of population admixture in the fastSTRUCTURE results, which are indicated by red arrows in Figures 6, 7.

### Modelling historical effective population size (*N*_e_) using genome-wide SNP data

**Figure 8:**
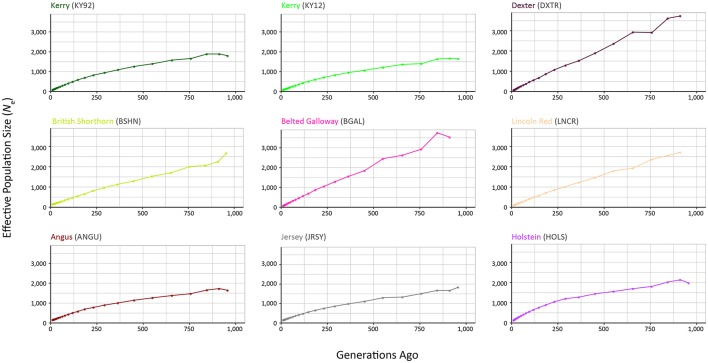
Genetic effective population size (*N*_e_) trends modelled using genome-wide SNP data. Results for the KY92 and KY12 populations are shown with seven comparator heritage and production cattle breeds.

The results from modelling historical *N*_e_ in a selection of production and heritage cattle breeds and populations (KY92, KY12, DXTR, BSHN, BGAL, LNCR, ANGU, JRSY, and HOLS) are provided in Supplementary Table 2 and visualised in Figure 8. The “demographic fingerprints” (Barbato et al., 2015) of the two Kerry populations shown in Figure 8 and tabulated in Supplementary Table 2 are more similar to those of the production breeds with large census populations (BSHN, ANGU, JRSY, HOLS) than the other heritage breeds with relatively small census population sizes (DXTR, BGAL, LNCR). The KY92, KY12, BSHN, ANGU, JRSY, and HOLS populations show a declining trend from historical *N*_e_ peaks between 1,500 and 2,000 more than 900 generations ago to *N*_e_ values estimated to be less than 200 within the last 20 generations. One the other hand, the DXTR, BSHN, BGAL and LNCR populations display a more severe decline from historical *N*_e_ peaks between 2,500 and 4,000 more than 900 generations ago to *N*_e_ values estimated to be less than 150 within the last 20 generations.

It is important to keep in mind these *N*_e_ trends may be partly a consequence of the relatively small sample sizes for the DXTR, BGAL, and LNCR breeds (see Table 2), coupled with different histories of migration, gene flow and, in particular, strong artificial selection in the production cattle populations. Notwithstanding these caveats, the most recent modelled *N*_e_ values for the KY92 and KY12 population samples are 89 and 88, respectively. These values are *N*_e_ estimates for 12 generations in the past and assuming a generation interval of between 4 and 6 years, which is based on a pedigree estimate from a similar heritage cattle population of 5.66 (Mészáros et al., 2015), this corresponds to between 48 and 72 years before 2012 (for the KY12 population). This is approximately the period between 1940 and 1965, which is during the time that the Kerry breed started to decline precipitously in census population size and also *N*_e_ estimated from herd book data (O'hUigín and Cunningham, 1990; Food and Agriculture Organization, 2007).

From a conservation perspective, livestock populations generally exhibit *N*_e_ values relative to total census population sizes (*N*_c_) that are substantially lower than seen in comparable wild mammal populations (Hall, 2016). Also, estimation of *N*_e_ using methods such as SNeP that leverage genome-wide SNP linkage disequilibrium (LD) data will tend to underestimate *N*_e_ because of physical linkage between many of the SNPs in the data set (Waples et al., 2016). Nevertheless, taking this into account, there is still cause for concern that the most recent *N*_e_ values modelled for the KY92 and KY12 population samples are below the critical *N*_e_ threshold of 100 recommended by Meuwissen (2009) for long-term viability of discrete livestock breeds and populations.

### Genomic relationship and analysis of inbreeding

Supplementary Table 1 shows a genomic relationship matrix in terms of genotype IBS for the genome-wide SNP data generated for individual animals in the KY92 and KY12 population samples. Close genomic relationship between individual animals sampled from the same herd is evident in the SNP genotype IBS values between samples. In addition, the relatively low genomic relationship between the KY12_06 and KY12_58 outlier samples (Figures 4–7) and the rest of the Kerry cattle sampled is also evident in Supplementary Table 1. These data emphasise the value of intrapopulation genomic relationship values for identifying animals (in this case, KY12_06 and KY12_58) that should not be used in breeding programmes. They also highlight the potential of genome-wide SNP data for providing a systematic approach to prioritising males and females with minimum genomic relationship for breeding to minimize loss of genetic diversity and maintain or increase *N*_e_ (Gandini et al., 2004; Meuwissen, 2009; de Cara et al., 2011, 2013).

**Figure 9:**
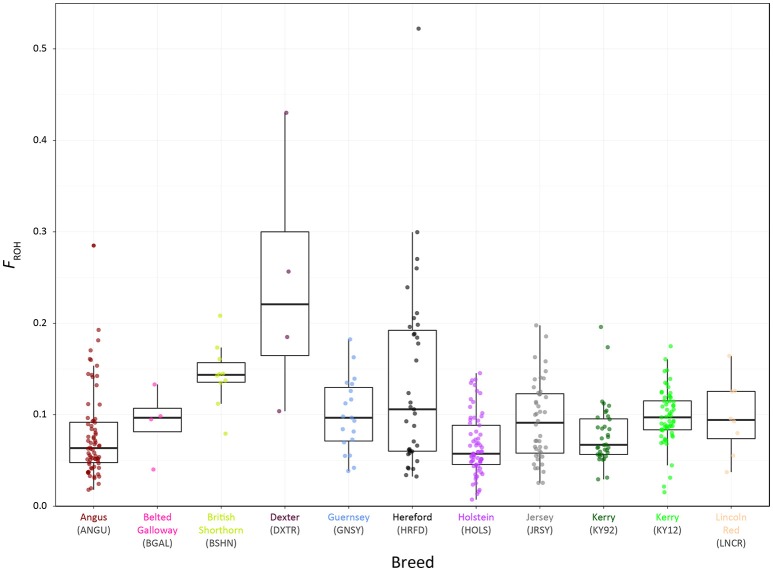
Tukey box plots showing the distributions of *F*_ROH_ values estimated with genome-wide SNP data for the KY92 and KY12 populations and nine comparator heritage and production cattle breeds.

Genome-wide autozygosity estimated from SNP data using *F*_ROH_ and the *F*_ROH_ statistic are visualised in Figure 9 for individual animals from the KY92 and KY12 population samples and a range of European comparator breeds. Additional summary ROH data is provided in Supplementary Table 3 and also Supplementary Figure 6, which reveals marked inter-population differences in ROH length and demonstrates that the SNP density of the Illumina® Bovine SNP50 BeadChip is too low to reliably capture ROH below 5 Mb in length, an observation previously reported by Purfield et al. (2012).

There is significant variation in *F*_ROH_ values among individual animals and between breeds and populations. The non-parametric Wilcoxon rank sum test was performed on *F*_ROH_ distributions for all pairwise population/breed comparisons with application of the Bonferroni correction *P*-value adjustment for multiple statistical tests (Supplementary Table 4). This analysis demonstrated that the KY12 population sample exhibited a significantly higher mean *F*_ROH_ value than the KY92 population sample (0.098 vs. 0.079; *P*_adjust_ = 0.0081). This is important from a conservation genetics perspective, indicating that genome-wide autozygosity, which is highly correlated with conventional pedigree-based estimates of inbreeding (*F*_PED_) for cattle (Purfield et al., 2012; Ferencaković et al., 2013; Martikainen et al., 2017), has increased for the Kerry cattle population in the 20 years between sampling of the KY92 and KY12 populations.

The importance of understanding and quantifying genome-wide autozygosity for genetic conservation purposes has recently been highlighted through correlation of *F*_ROH_ with inbreeding depression for a range of production traits in domestic cattle (Bjelland et al., 2013; Pryce et al., 2014; Kim et al., 2015). Importantly, *F*_ROH_ has also been shown to correlate with inbreeding depression for bovine fertility traits in both males (Ferencaković et al., 2017) and females (Kim et al., 2015; Martikainen et al., 2017). Finally, according to basic population genetic principles, recent inbreeding captured by *F*_ROH_ will lead to recessive deleterious genomic variants emerging at a population level—a phenomenon that has been studied in both humans and cattle (Szpiech et al., 2013; Zhang et al., 2015).

### Genome-wide signatures of selection in the kerry cattle breed

**Figure 10:**
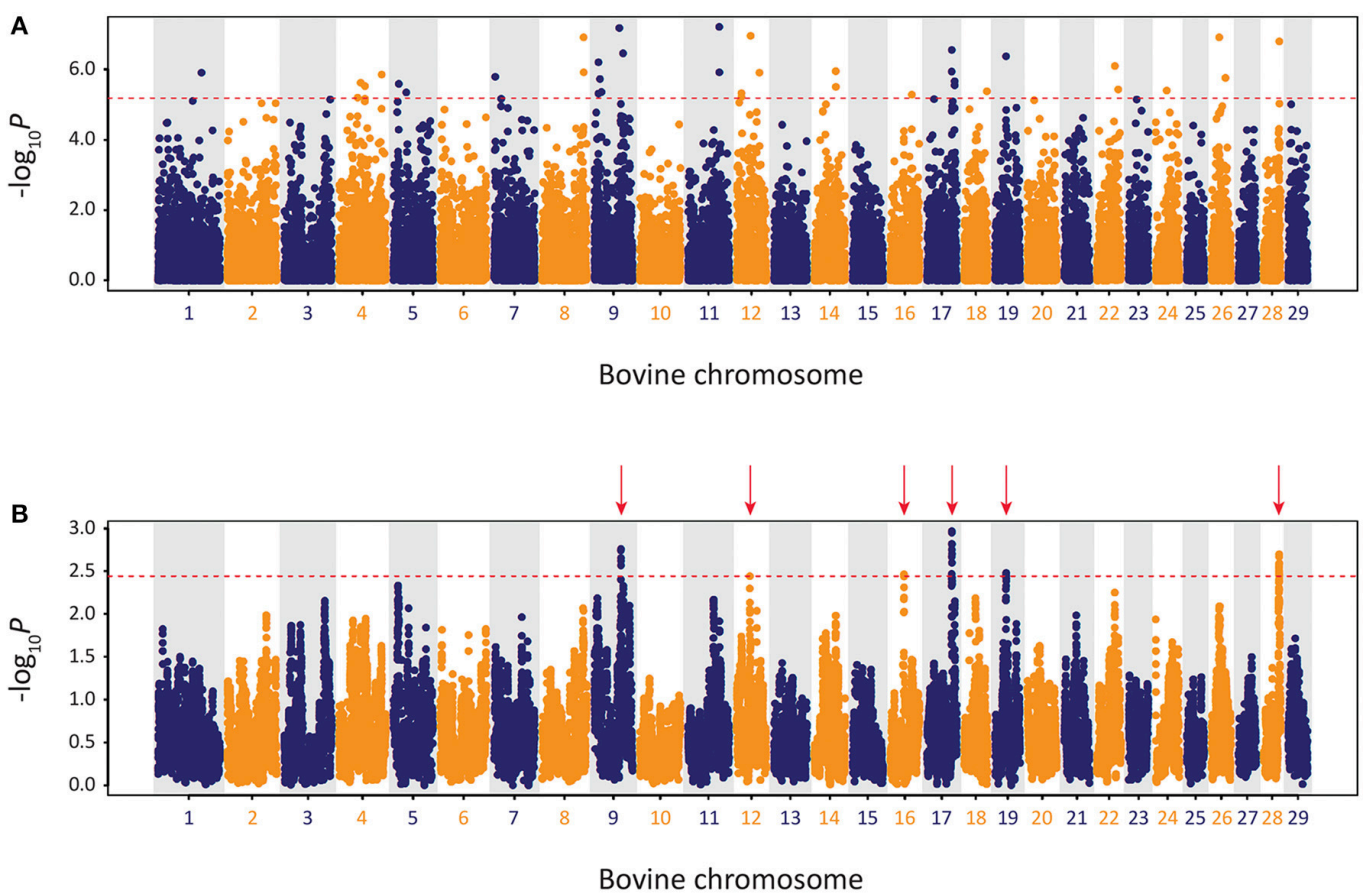
Manhattan plots of composite selection signal (CSS) results for Kerry cattle (*n* = 98) contrasted with EU cattle (*n* = 102). **(A)** Unsmoothed results. **(B)** Smoothed results obtained by averaging CSS of SNPs within each 1 Mb window. Red dotted line on each plot denotes the genome-wide 0.1% threshold for the empirical CSS scores. Red vertical arrows indicate selection peaks detected on BTA09, BTA12, BTA16, BTA17, BTA19, and BTA28.

The results of the genome-wide scan for signatures of selection using the CSS method in the Kerry cattle breed are shown in Figure 10. Six distinct selection signatures were detected on BTA9, BTA12, BTA16, BTA17, BTA19, and BTA28. A total of 178 genes were located within the genomic ranges ± 1.0 Mb of selection peaks and 32 of these genes were located within the boundaries of a selection peak. Supplementary Table 5 provides detailed information for these 178 genes.

A single gene was located within the BTA9 selection peak—the phosphodiesterase 7B gene (*PDE7B*), which has been associated with neurobiological processes (de Gortari and Mengod, 2010) and has been previously linked to genetic changes associated with dog (*Canis lupus familiaris*) domestication and behaviour (Freedman et al., 2016). A single gene was also located within the BTA16 selection peak—the dorsal inhibitory axon guidance protein gene (*DRAXIN*), which encodes a protein that regulates axon guidance, neural circuit formation and vertebrate brain development (Islam et al., 2009; Shinmyo et al., 2015). Twenty-four genes were located within the BTA17 selection peak, including *BICDL1, RAB35*, and *RNF10*, which have been associated with neurobiology and brain development (Hoshikawa et al., 2008; Schlager et al., 2010; Villarroel-Campos et al., 2016) and *SIRT4* and *COQ5* that function in cellular metabolism (Kawamukai, 2015; Elkhwanky and Hakkola, 2017). Six genes were located within the BTA28 selection peak, including, most notably, the Rho GTPase activating protein 22 gene (*ARHGAP22*), which has recently been associated with bovine fertility as an mRNA expression biomarker for oocyte competence in cumulus cells (Melo et al., 2017).

To obtain a broader perspective on natural and artificial selection acting at a population level on the Kerry cattle genome, a functional gene set enrichment approach (GSEA) was taken using IPA with the 178 genes located within ± 1.0 Mb of each selection peak (Supplementary Table 5). Of these 178 genes, 141 could be mapped to the IPA knowledgebase and the summary results for the IPA *Physiological System Development and Function* category are shown in Supplementary Table 6, revealing an enrichment of biological processes associated with nervous system development and behaviour. This functional enrichment coupled with the neurobiologically relevant single-gene selection peaks on BTA9 (*PDE7B*) and BTA16 (*DRAXIN*) suggests that natural and/or artificial selection related to brain development and behaviour has been important in the microevolution of the Kerry cattle breed. In this regard, it is therefore noteworthy that Kerry cattle, including bulls, are recognised as being comparatively docile and easy to manage (Curran, 1990).

### Genomics, genetic distinctiveness and microevolution of kerry cattle: implications for breed management and genetic conservation

The genome-wide phylogenetic and population genetic analyses detailed here demonstrate that Kerry cattle represent an important farm animal genetic resource, befitting the breed's status as a livestock population with a unique history of adaptation to the climate and physical geography of southwest Ireland at the edge of Western Europe. Notably, from a genetic conservation and breed management perspective, high-resolution comparative PCA (Figures 4, 5) and genetic clustering results (Figures 6, 7) demonstrate that Kerry cattle are markedly distinct from other British and European cattle populations. This observation may also be placed in the context of recent paleogenomic studies that have detected ancient gene flow from wild British aurochs (*B. primigenius*) into the ancestors of present-day Kerry cattle (Orlando, 2015; Park et al., 2015; Upadhyay et al., 2017).

The current genetic status of the Kerry cattle population is underlined by analyses of genetic effective population size (*N*_e_) and inbreeding using genome-wide SNP data. As shown in Table 2, genome-wide observed heterozygosity (*H*_o_) is relatively high in the KY92 and K12 population samples, particularly for endangered heritage cattle breeds. However, it has been long recognised that monitoring *N*_e_ is a more important tool for rational breed management and long-term conservation of endangered livestock populations (Notter, 1999; Gandini et al., 2004; Biscarini et al., 2015). As shown in Figure 8 and Supplementary Table 2, the Kerry cattle population has a recent demographic trend of *N*_e_ decline, to the point where the most recent modelled *N*_e_ values are below the recommended threshold for sustainable breed management and conservation (Meuwissen, 2009). There is also cause for concern that genomic inbreeding estimated using genome-wide autozygosity (*F*_ROH_) and visualised in Figure 9 has increased significantly in the 20-year period between the sampling of the KY92 and KY12 Kerry cattle populations.

In a more positive light, as shown in the present study, detection of discrete signatures of selection using the relatively low-density Illumina® Bovine SNP50 BeadChip is encouraging for wider studies of genome-wide microevolution in endangered heritage livestock populations such as Kerry cattle. Future surveys of heritage livestock populations that use higher-density SNP array platforms and ultimately whole-genome sequence data could provide exquisitely detailed information on the genomic regions and associated polygenic production, health, fertility and behavioural traits shaped, over many centuries, by the agro-ecology and pre-industrial farming systems of southwest Ireland.

In conclusion, the results presented here for the Kerry cattle population demonstrate that population genomics analyses of large SNP data sets can provide useful information concerning the microevolution and recent genetic history of heritage livestock breeds. In particular, we would recommend that comparable surveys in other populations consider the use of genome-wide scans for signatures of selection, which can provide a functional genomics perspective on evolutionary adaptations to particular agricultural environments and production systems.

## Data accessibility

The Illumina® Bovine SNP50 BeadChip SNP genotype data for the Kerry cattle KY92 and KY12 population samples generated for this study are available from the Dryad Digital Repository: https://doi.org/10.5061/dryad.8fk81.

## Ethics statement

With the exception of Kerry cattle sampled during 2011–12, all samples and data was obtained from previously published scientific studies. The re-use of these samples and data is consistent with the 3Rs principles on replacement, refinement and reduction of animals in research (www.nc3rs.org.uk/the-3rs). For the 2011-12 Kerry cattle, population owners' consent to sample DNA for research was obtained and individual owners conducted sampling of animals using non-invasive nasal swabs. In this regard, scientific animal protection in Ireland is subject to European Union Directive 2010/63/EU, which states that the Directive does not apply to “practices not likely to cause pain, suffering, distress or lasting harm equivalent to, or higher than, that caused by the introduction of a needle in accordance with good veterinary practice.”

## Author contributions

DEM, DAM, AF, and JK conceived and designed the project; DEM, IWR, DAM, AF, and JK organised sample collection and genotyping; SB, GM, IWR, DAM, SP, CC, IASR, and DEM performed the analyses; SB and DEM wrote the manuscript and all authors reviewed and approved the final manuscript.

### Conflict of interest statement

The authors IWR and SP are employed by IdentiGEN, Ltd. The other authors declare that the research was conducted in the absence of any commercial or financial relationships that could be construed as a potential conflict of interest.
