# Supplementary material for: Genomic Characterisation of the Indigenous Irish Kerry Cattle Breed
Source: Front Genet. 2018 Feb 19;9:51. doi: 10.3389/fgene.2018.00051 (PMC5827531; doi:10.3389/fgene.2018.00051)
Supplement: Supplementary file 3 [file DataSheet1.PDF]

Frontiers in Genetics Original Research

Supplementary Material

Genomic characterisation of the indigenous Irish Kerry cattle breed

Sam Browett, Gillian McHugo, Ian W. Richardson, David A. Magee, Stephen D. E. Park, Alan G. Fahey, John F. Kearney, Carolina N. Correia, Imtiaz A. S. Randhawa, David E. MacHugh*

*** Correspondence:** David E. MacHugh, Animal Genomics Laboratory, UCD School of

Agriculture and Food Science, University College Dublin, Belfield, Dublin, D04 V1W8, Ireland.

Email: david.machugh@ucd.ie

1 Supplementary Figures and Tables

1.1. Supplementary Figures

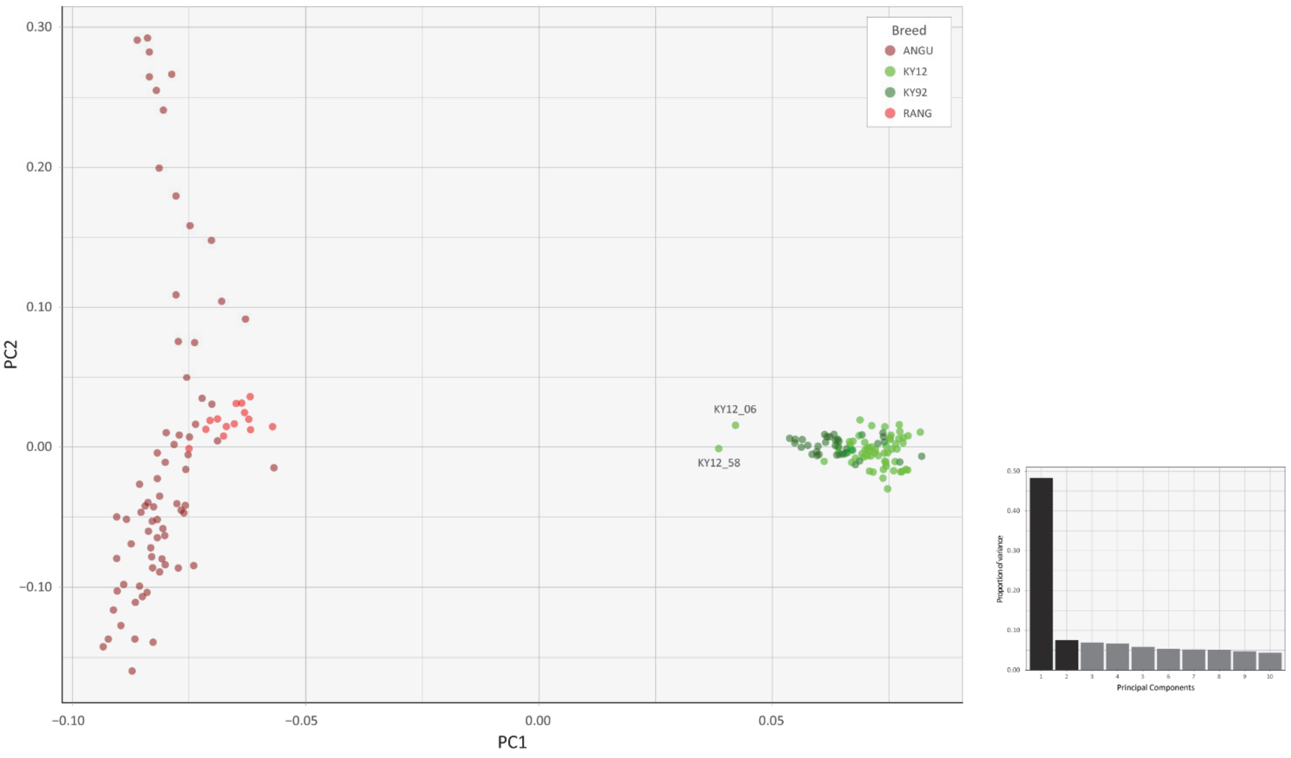

Supplementary Figure 1. Principal component analysis plot constructed for PC1 and PC2 from genome-wide SNP data (37,395 autosomal SNPs) for the ANGU, RANG, KY92 and KY12 data set of 184 individual animals. The smaller histogram plot shows the relative variance contributions for the first 10 PCs and PC1 and PC2 account for 48.3% and 7.5% of the total variation for PC1–10, respectively.

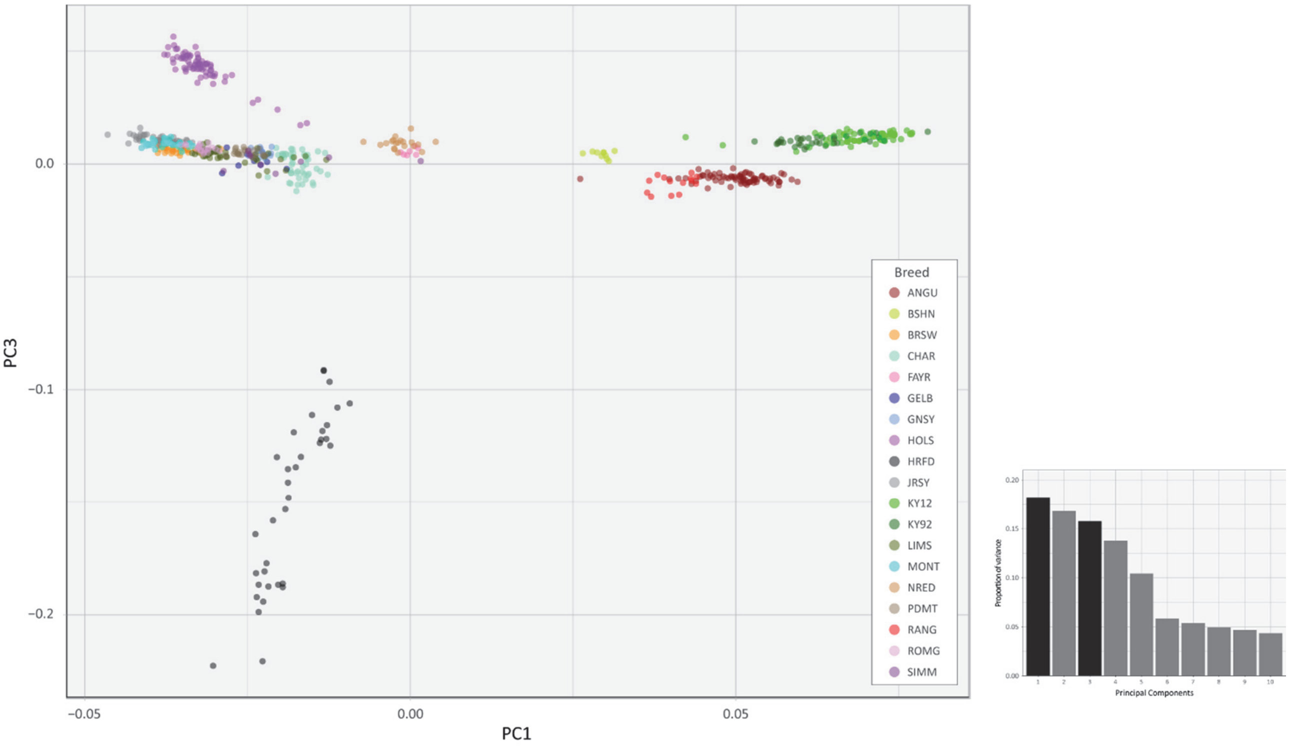

Supplementary Figure 2. Principal component analysis plot constructed for PC1 and PC3 from genome-wide SNP data (36,621 autosomal SNPs) for the EU data set of 605 individual animals. The smaller histogram plot shows the relative variance contributions for the first 10 PCs and PC1 and PC3 account for 18.2% and 15.7% of the total variation for PC1–10, respectively.

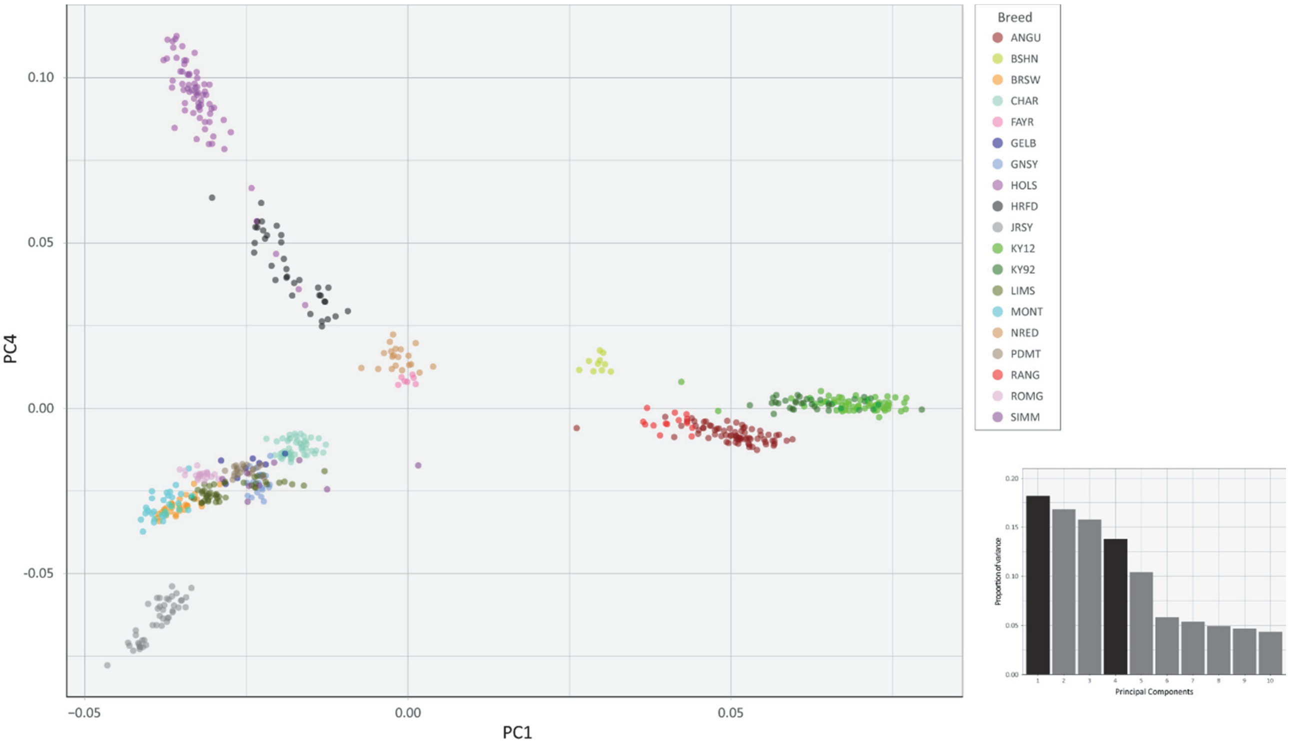

Supplementary Figure 3. Principal component analysis plot constructed for PC1 and PC4 from genome-wide SNP data (36,621 autosomal SNPs) for the EU data set of 605 individual animals. The smaller histogram plot shows the relative variance contributions for the first 10 PCs and PC1 and PC4 account for 18.2% and 13.8% of the total variation for PC1–10, respectively.

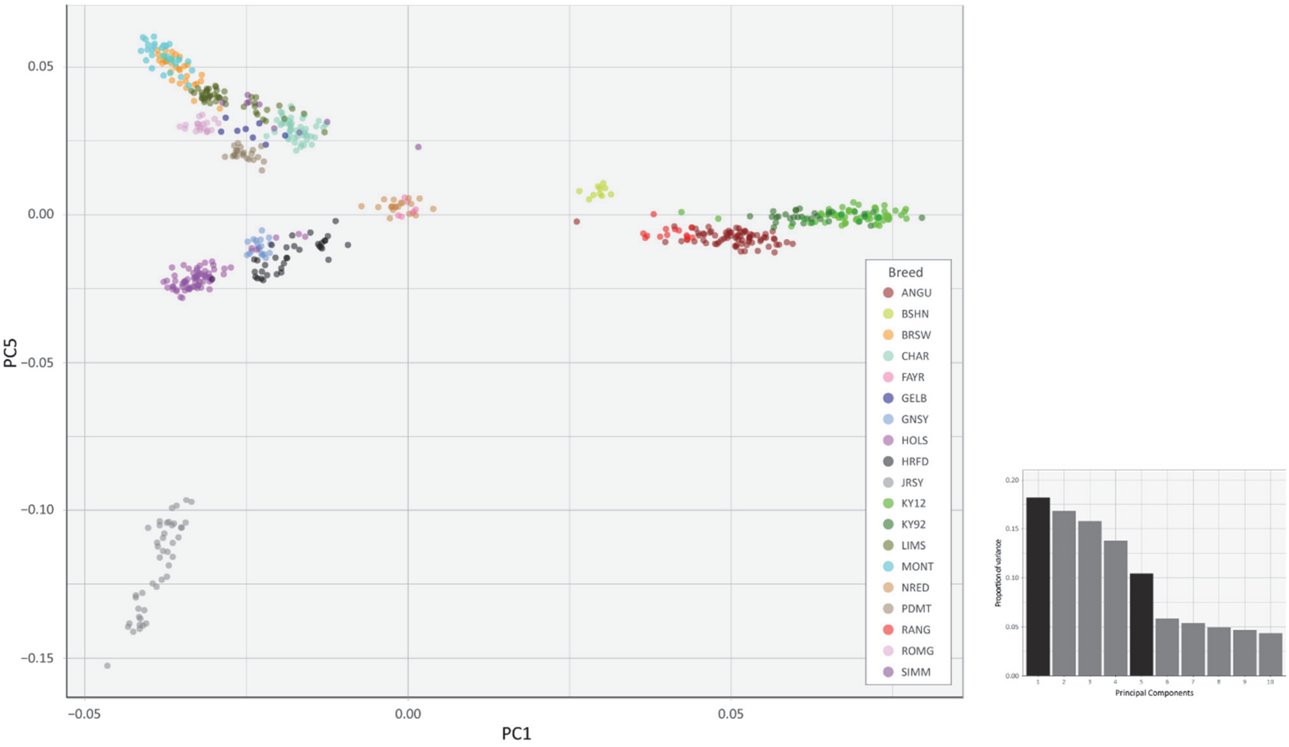

Supplementary Figure 4. Principal component analysis plot constructed for PC1 and PC5 from genome-wide SNP data (36,621 autosomal SNPs) for the EU data set of 605 individual animals. The smaller histogram plot shows the relative variance contributions for the first 10 PCs and PC1 and PC5 account for 18.2% and 10.4% of the total variation for PC1–10, respectively.

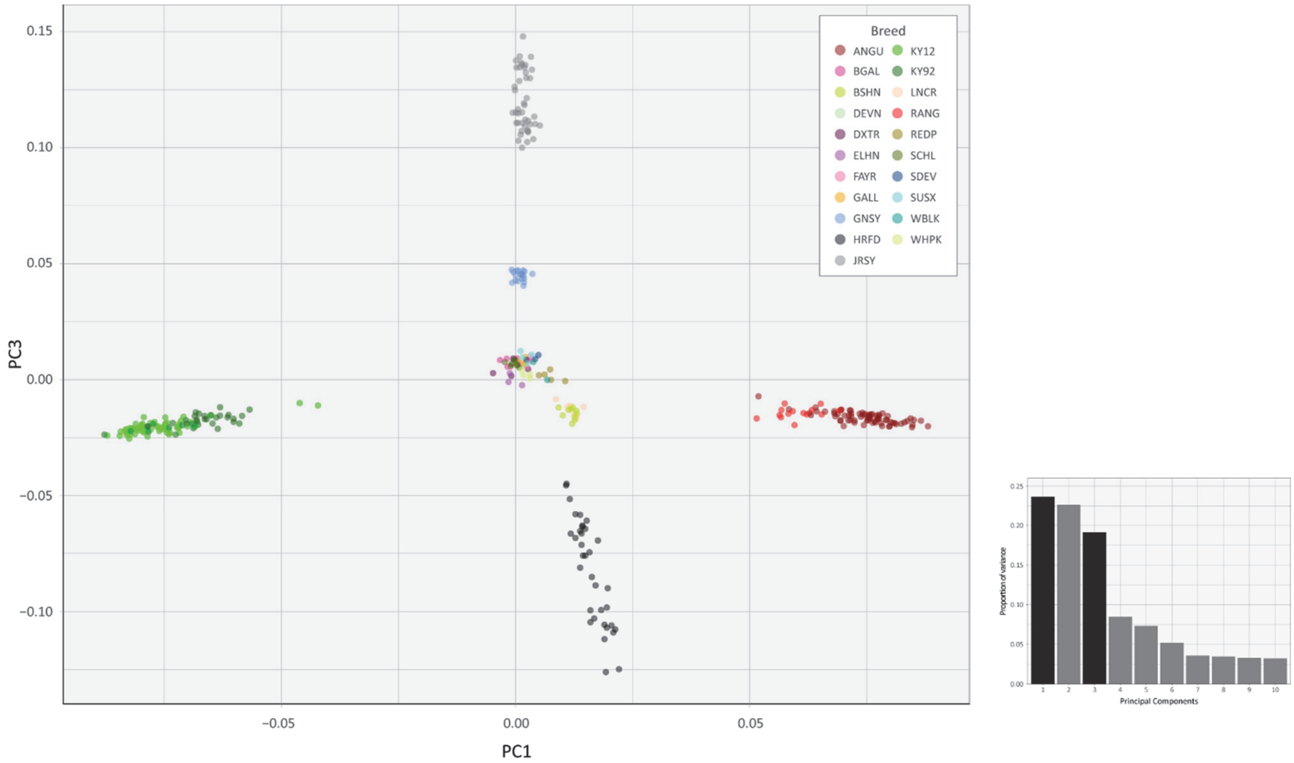

Supplementary Figure 5. Principal component analysis plot constructed for PC1 and PC3 from genome-wide SNP data (37,395 autosomal SNPs) for the BI data set of 351 individual animals. The smaller histogram plot shows the relative variance contributions for the first 10 PCs and PC1 and PC3 account for 23.7% and 19.1% of the total variation for PC1–10, respectively.

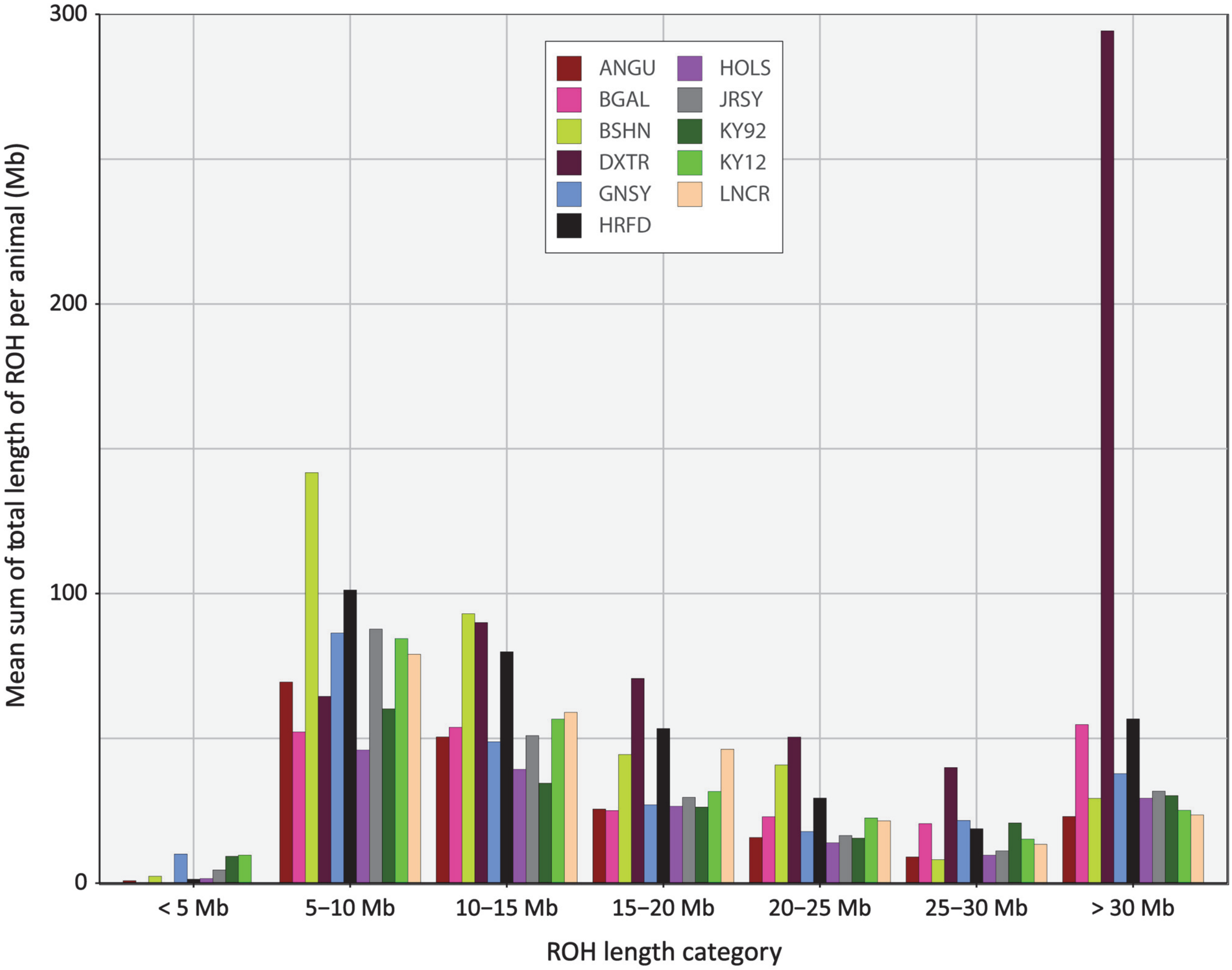

Supplementary Figure 6. Distribution of runs of homozygosity (ROH) by size for individual animals by breed. For each breed, ROH were divided into length categories (Mb), and a mean length was calculated for the proportion of the genome covered by ROH of that size.

1.2. Supplementary Tables

Supplementary Table 1: Identity-by-state (IBS) values for KY92 (*n* = 36) and KY12 (*n* = 62) Kerry cattle samples (Excel file - **Supp_table_01.xlsx**).

Supplementary Table 2: Historical effective population size (N_e) values estimated with the SNeP software package.

Gens Ago	KY92 N_e	KY12 N_e	DXTR N_e	BSHN N_e	BGAL N_e	LNCR N_e	ANGU N_e	JRSY N_e	HOLS N_e
**12**	89	88	67	153	72	97	162	153	141
**14**	96	94	78	159	85	105	166	159	152
**16**	105	101	88	166	95	114	173	168	165
**19**	116	110	103	174	108	126	181	177	182
**22**	129	122	118	182	128	143	194	187	202
**26**	145	135	137	190	144	157	205	202	223
**31**	166	152	162	209	168	174	222	215	251
**37**	190	172	195	223	203	194	241	240	285
**44**	216	195	230	246	229	222	267	260	321
**53**	250	227	273	273	267	259	293	290	365
**65**	297	264	325	301	325	293	333	321	423
**79**	352	311	380	352	384	348	380	373	489
**97**	415	363	477	405	469	407	436	424	569
**120**	496	436	567	486	573	498	511	481	659
**149**	591	515	681	552	692	578	590	567	767
**186**	695	607	867	662	877	716	700	653	894
**233**	826	716	1,079	816	1,056	856	786	753	1,055
**293**	948	827	1,297	970	1,295	1,021	905	866	1,204
**365**	1,096	958	1,524	1,131	1,557	1,229	1,012	986	1,280
**453**	1,264	1,067	1,899	1,293	1,846	1,469	1,149	1,113	1,444
**552**	1,398	1,216	2,367	1,528	2,445	1,798	1,269	1,299	1,557
**657**	1,583	1,365	2,932	1,705	2,621	1,934	1,384	1,334	1,698
**758**	1,662	1,403	2,914	2,012	2,925	2,362	1,479	1,510	1,804
**846**	1,888	1,638	3,613	2,061	3,748	2,554	1,660	1,676	2,022
**912**	1,889	1,667	3,735	2,247	3,525	2,718	1,732	1,668	2,134
**958**	1,798	1,654	---	2,677	---	---	1,644	1,840	1,972

Supplementary Table 3: Results from runs of homozygosity (ROH) analysis in various European cattle breeds/populations. Individual mean values shown ± standard error (SE).

Breed/population code	Mean number ROH segments ± SE	Mean ROH length (kb) ± SE	Mean F_{ROH} ± SE	Median F_{ROH}
ANGU	16.78 ± 0.88	11,128 ± 269	0.078 ± 0.006	0.064
BGAL	16.50 ± 2.47	13,405 ± 1,146	0.092 ± 0.019	0.097
BSHN	32.40 ± 1.55	11,022 ± 514	0.144 ± 0.011	0.144
DXTR	29.50 ± 5.85	19,959 ± 2,574	0.244 ± 0.069	0.221
GNSY	22.58 ± 1.66	10,806 ± 406	0.100 ± 0.009	0.097
HRFD	26.51 ± 2.57	11,988 ± 459	0.136 ± 0.017	0.106
HOLS	13.00 ± 0.56	12,366 ± 351	0.066 ± 0.004	0.057
JRSY	20.77 ± 1.03	10,786 ± 333	0.093 ± 0.007	0.091
KY92	17.06 ± 0.80	11,337 ± 387	0.079 ± 0.006	0.067
KY12	22.77 ± 0.67	10,609 ± 223	0.098 ± 0.004	0.097
LNCR	19.75 ± 2.48	11,990 ± 562	0.097 ± 0.015	0.094

Supplementary Table 4: Adjusted *P* values obtained from Wilcoxon signed-rank test results for pairwise comparisons of F_{ROH} distributions for the KY92 and KY12 populations and European comparator breeds. The Bonferroni correction was applied to correct for multiple statistical tests and P_{adjust} values ≤ 0.05 are highlighted.

Breed	ANGU	BGAL	BSHN	DXTR	GNSY	HRFD	HOLS	JRSY	KY92	KY12	LNCR
ANGU											
BGAL	1.0000										
BSHN	0.0078	1.0000									
DXTR	0.1790	1.0000	1.0000								
GNSY	0.6204	1.0000	0.3665	0.6584							
HRFD	0.0916	1.0000	1.0000	1.0000	1.0000						
HOLS	1.0000	1.0000	0.0003	0.1099	0.0856	0.0078					
JRSY	1.0000	1.0000	0.0404	0.3443	1.0000	1.0000	0.0668				
KY92	1.0000	1.0000	0.0008	0.0457	1.0000	1.0000	1.0000	1.0000			
KY12	0.0008	1.0000	0.0244	0.4638	1.0000	1.0000	0.0000	1.0000	0.0081		
LNCR	1.0000	1.0000	1.0000	1.0000	1.0000	1.0000	1.0000	1.0000	1.0000	1.0000	

Supplementary Table 5: Bovine genes located within the genomic ranges ± 1.0 Mb of selection peaks detected using the CSS selection scan method for Kerry cattle (*n* = 98) compared to composite EU breed population (*n* = 102).

Chromosome	Start position	End position	Bovine Ensembl ID	Gene symbol	Position relative to selection peak
BTA09	73,977,645	74,002,359	ENSBTAG00000007975	*ALDH8A1*	Upstream
BTA09	74,011,379	74,096,501	ENSBTAG00000020174	*HBS1L*	Upstream
BTA09	74,021,313	74,021,407	ENSBTAG00000047686	Novel gene	Upstream
BTA09	74,222,882	74,258,203	ENSBTAG00000012074	*MYB*	Upstream
BTA09	74,330,947	74,544,060	ENSBTAG00000017958	*AHI1*	Upstream
BTA09	74,338,936	74,339,254	ENSBTAG00000046713	Novel gene	Upstream
BTA09	74,453,535	74,454,206	ENSBTAG00000046495	Novel gene	Upstream
BTA09	74,586,367	74,586,481	ENSBTAG00000028919	*5S_rRNA*	Upstream
BTA09	75,034,593	75,191,797	ENSBTAG00000015888	*PDE7B*	Within peak
BTA09	75,236,009	75,254,523	ENSBTAG00000007799	*MTFR2*	Downstream
BTA09	75,262,831	75,291,455	ENSBTAG00000007802	*BCLAF1*	Downstream
BTA09	75,308,953	75,309,469	ENSBTAG00000046168	Novel gene	Downstream
BTA09	75,364,392	75,519,232	ENSBTAG00000001598	*MAP7*	Downstream
BTA09	75,549,283	75,778,418	ENSBTAG00000009065	*MAP3K5*	Downstream
BTA09	75,813,334	75,891,921	ENSBTAG00000016791	*PEX7*	Downstream
BTA09	75,897,878	75,900,146	ENSBTAG00000016793	*SLC35D3*	Downstream
BTA09	75,979,324	76,012,398	ENSBTAG00000015638	*IL20RA*	Downstream
BTA09	76,064,702	76,079,984	ENSBTAG00000017000	*IL22RA2*	Downstream
BTA09	76,092,204	76,116,593	ENSBTAG00000012544	*IFNGR1*	Downstream
BTA12	44,295,888	44,616,940	ENSBTAG00000008647	*KLHL1*	Upstream
BTA12	44,321,995	44,322,044	ENSBTAG00000046715	*U6*	Upstream
BTA12	44,822,977	44,823,477	ENSBTAG00000046942	Novel gene	Upstream
BTA12	44,865,039	44,865,164	ENSBTAG00000042849	*U6atac*	Downstream
BTA16	41,985,339	42,040,600	ENSBTAG00000024493	*DHRS3*	Upstream
BTA16	42,082,188	42,349,474	ENSBTAG00000016080	*VPS13D*	Upstream

Chromosome	Start position	End position	Bovine Ensembl ID	Gene symbol	Position relative to selection peak
BTA16	42,084,905	42,085,048	ENSBTAG00000044521	*ACA59*	Upstream
BTA16	42,376,258	42,409,768	ENSBTAG00000024928	*TNFRSF1B*	Upstream
BTA16	42,440,211	42,493,773	ENSBTAG00000039937	*TNFRSF8*	Upstream
BTA16	42,538,502	42,548,038	ENSBTAG00000019556	*MIIP*	Upstream
BTA16	42,561,715	42,581,003	ENSBTAG00000005314	*MFN2*	Upstream
BTA16	42,595,152	42,625,615	ENSBTAG00000002052	*PLOD1*	Upstream
BTA16	42,633,976	42,634,091	ENSBTAG00000045716	*U5*	Upstream
BTA16	42,648,166	42,653,791	ENSBTAG00000019388	*KIAA2013*	Upstream
BTA16	42,700,409	42,701,796	ENSBTAG00000021739	*NPPB*	Upstream
BTA16	42,712,044	42,713,502	ENSBTAG00000006709	*NPPA*	Upstream
BTA16	42,719,367	42,750,151	ENSBTAG00000020700	*CLCN6*	Upstream
BTA16	42,750,520	42,765,210	ENSBTAG00000020698	*MTHFR*	Upstream
BTA16	42,765,851	42,788,588	ENSBTAG00000009386	*C1orf167*	Upstream
BTA16	42,806,430	42,825,905	ENSBTAG00000009384	*AGTRAP*	Upstream
BTA16	42,840,335	42,868,899	ENSBTAG00000014359	*DRAXIN*	Within peak
BTA16	42,876,001	42,881,565	ENSBTAG00000013275	*MAD2L2*	Downstream
BTA16	42,881,664	42,899,260	ENSBTAG00000014777	*FBXO6*	Downstream
BTA16	42,901,488	42,905,541	ENSBTAG00000032617	*FBXO44*	Downstream
BTA16	42,906,700	42,912,737	ENSBTAG00000007223	*FBXO2*	Downstream
BTA16	43,027,923	43,064,263	ENSBTAG00000015533	*DISP3*	Downstream
BTA16	43,258,416	43,268,285	ENSBTAG00000014014	*UBIAD1*	Downstream
BTA16	43,275,756	43,396,218	ENSBTAG00000015325	*MTOR*	Downstream
BTA16	43,328,466	43,334,550	ENSBTAG00000015340	*ANGPTL7*	Downstream
BTA16	43,403,640	43,426,116	ENSBTAG00000002590	*EXOSC10*	Downstream
BTA16	43,445,563	43,449,438	ENSBTAG00000005304	*SRM*	Downstream
BTA16	43,463,199	43,478,362	ENSBTAG00000012808	*MASP2*	Downstream
BTA16	43,475,060	43,486,514	ENSBTAG00000003697	*TARDBP*	Downstream

Chromosome	Start position	End position	Bovine Ensembl ID	Gene symbol	Position relative to selection peak
BTA16	43,786,499	43,839,928	ENSBTAG00000019818	*CASZ1*	Downstream
BTA16	43,848,339	43,992,183	ENSBTAG00000013538	*PEX14*	Downstream
BTA17	63,448,841	63,466,323	ENSBTAG00000011930	*DDX54*	Upstream
BTA17	63,466,650	63,472,165	ENSBTAG00000011937	*RITA1*	Upstream
BTA17	63,474,993	63,497,850	ENSBTAG00000001806	*IQCD*	Upstream
BTA17	63,528,831	63,558,939	ENSBTAG00000011102	*TPCN1*	Upstream
BTA17	63,561,392	63,591,370	ENSBTAG00000011103	*SLC8B1*	Upstream
BTA17	63,611,774	63,640,159	ENSBTAG00000014628	*OAS2*	Upstream
BTA17	63,638,182	63,646,117	ENSBTAG00000039861	*OAS1Y*	Upstream
BTA17	63,654,698	63,678,163	ENSBTAG00000037527	*OAS1X*	Upstream
BTA17	63,682,758	63,748,775	ENSBTAG00000004247	*RPH3A*	Upstream
BTA17	64,010,592	64,056,567	ENSBTAG00000002048	*PTPN11*	Upstream
BTA17	64,051,485	64,051,591	ENSBTAG00000042345	*U6*	Upstream
BTA17	64,115,683	64,120,590	ENSBTAG00000031723	*RPL6*	Upstream
BTA17	64,226,562	64,226,709	ENSBTAG00000043928	*SNORA79*	Upstream
BTA17	64,230,116	64,247,469	ENSBTAG00000010958	Novel gene	Upstream
BTA17	64,247,473	64,279,571	ENSBTAG00000046730	Novel gene	Upstream
BTA17	64,291,518	64,308,778	ENSBTAG00000008762	Novel gene	Upstream
BTA17	64,313,879	64,328,239	ENSBTAG00000008761	*TRAFD1*	Upstream
BTA17	64,361,788	64,413,468	ENSBTAG00000006666	*NAA25*	Upstream
BTA17	64,419,027	64,426,503	ENSBTAG00000006665	*ERP29*	Upstream
BTA17	64,436,074	64,463,404	ENSBTAG00000006659	*TMEM116*	Within peak
BTA17	64,436,918	64,437,022	ENSBTAG00000043619	*U6*	Within peak
BTA17	64,491,726	64,494,161	ENSBTAG00000031693	*ADAM1B*	Within peak
BTA17	64,508,406	64,530,237	ENSBTAG00000009346	*MAPKAPK5*	Within peak
BTA17	64,551,612	64,577,901	ENSBTAG00000008743	*ALDH2*	Within peak
BTA17	64,643,561	64,722,444	ENSBTAG00000003696	*BICDL1*	Within peak

Chromosome	Start position	End position	Bovine Ensembl ID	Gene symbol	Position relative to selection peak
BTA17	64,724,244	64,742,928	ENSBTAG00000022044	*RAB35*	Within peak
BTA17	64,752,926	64,803,078	ENSBTAG00000017379	*GCN1*	Within peak
BTA17	64,790,997	64,791,128	ENSBTAG00000042215	*U4*	Within peak
BTA17	64,809,254	64,813,289	ENSBTAG00000017389	*RPLP0*	Within peak
BTA17	64,818,100	64,833,437	ENSBTAG00000018108	*PXN*	Within peak
BTA17	64,874,908	64,875,048	ENSBTAG00000048167	*U4*	Within peak
BTA17	64,875,357	64,891,606	ENSBTAG00000021168	*SIRT4*	Within peak
BTA17	64,875,706	64,875,846	ENSBTAG00000046842	*U4*	Within peak
BTA17	64,899,652	64,902,627	ENSBTAG00000026732	Novel gene	Within peak
BTA17	64,910,531	64,913,110	ENSBTAG00000047084	Novel gene	Within peak
BTA17	64,913,867	64,937,017	ENSBTAG00000009800	*MSI1*	Within peak
BTA17	64,995,248	64,997,121	ENSBTAG00000012788	*COX6A1*	Within peak
BTA17	64,998,266	65,000,579	ENSBTAG00000012790	*TRIAP1*	Within peak
BTA17	65,000,550	65,007,056	ENSBTAG00000012791	*GATC*	Within peak
BTA17	65,008,122	65,014,401	ENSBTAG00000012792	*SRSF9*	Within peak
BTA17	65,042,590	65,045,024	ENSBTAG00000024605	*DYNLL1*	Within peak
BTA17	65,047,403	65,064,488	ENSBTAG00000015761	*COQ5*	Within peak
BTA17	65,067,805	65,101,419	ENSBTAG00000015767	*RNF10*	Within peak
BTA17	65,102,638	65,104,915	ENSBTAG00000005385	*POP5*	Downstream
BTA17	65,154,254	65,170,682	ENSBTAG00000020049	*CABP1*	Downstream
BTA17	65,184,868	65,194,879	ENSBTAG00000020050	*MLEC*	Downstream
BTA17	65,206,692	65,215,132	ENSBTAG00000007483	*UNC119B*	Downstream
BTA17	65,220,605	65,237,484	ENSBTAG00000007484	*ACADS*	Downstream
BTA17	65,280,174	65,310,143	ENSBTAG00000016338	*SPPL3*	Downstream
BTA17	65,369,277	65,369,579	ENSBTAG00000045977	*Metazoa_SRP*	Downstream
BTA17	65,417,479	65,434,194	ENSBTAG00000021795	*HNF1A*	Downstream
BTA17	65,435,169	65,445,334	ENSBTAG00000021796	*C12orf43*	Downstream

Chromosome	Start position	End position	Bovine Ensembl ID	Gene symbol	Position relative to selection peak
BTA17	65,446,987	65,463,409	ENSBTAG00000003297	*OASL*	Downstream
BTA17	65,456,341	65,456,721	ENSBTAG00000046887	Novel gene	Downstream
BTA17	65,508,336	65,514,043	ENSBTAG00000032047	Novel gene	Downstream
BTA17	65,519,357	65,552,128	ENSBTAG00000014376	*ANKRD13A*	Downstream
BTA17	65,556,431	65,596,948	ENSBTAG00000006506	*GIT2*	Downstream
BTA17	65,602,269	65,616,737	ENSBTAG00000006504	*TCHP*	Downstream
BTA17	65,628,780	65,651,239	ENSBTAG00000002591	*GLTP*	Downstream
BTA17	65,678,454	65,699,847	ENSBTAG00000000031	*TRPV4*	Downstream
BTA17	65,711,149	65,713,710	ENSBTAG00000031993	*FAM222A*	Downstream
BTA17	65,863,712	65,882,658	ENSBTAG00000005183	*MVK*	Downstream
BTA17	65,882,910	65,892,166	ENSBTAG00000005178	*MMAB*	Downstream
BTA17	65,905,227	65,944,588	ENSBTAG00000002698	*UBE3B*	Downstream
BTA17	65,950,166	65,979,498	ENSBTAG00000002697	*KCTD10*	Downstream
BTA17	65,981,418	66,022,389	ENSBTAG00000002695	*MYO1H*	Downstream
BTA19	29,518,150	29,572,290	ENSBTAG00000000120	*USP43*	Upstream
BTA19	29,581,010	29,594,334	ENSBTAG00000005048	*DHRS7C*	Upstream
BTA19	29,597,619	29,605,540	ENSBTAG00000005051	*GSG1L2*	Upstream
BTA19	29,608,715	29,648,238	ENSBTAG00000005052	*GLP2R*	Upstream
BTA19	29,653,157	29,661,163	ENSBTAG00000025088	*RCVRN*	Upstream
BTA19	29,670,744	29,876,650	ENSBTAG00000019107	*GAS7*	Upstream
BTA19	29,708,266	29,708,364	ENSBTAG00000043853	*5S_rRNA*	Upstream
BTA19	29,956,798	30,003,344	ENSBTAG00000025337	Novel gene	Upstream
BTA19	30,031,040	30,059,203	ENSBTAG00000009702	*MYH8*	Upstream
BTA19	30,080,604	30,103,436	ENSBTAG00000037794	*MYH4*	Upstream
BTA19	30,110,728	30,134,757	ENSBTAG00000018204	*MYH1*	Upstream
BTA19	30,137,767	30,165,109	ENSBTAG00000007090	*MYH2*	Upstream
BTA19	30,230,160	30,251,091	ENSBTAG00000011803	*MYH3*	Upstream

Chromosome	Start position	End position	Bovine Ensembl ID	Gene symbol	Position relative to selection peak
BTA19	30,276,156	30,296,404	ENSBTAG00000021780	*SCO1*	Upstream
BTA19	30,296,519	30,303,510	ENSBTAG00000046919	*ADPRM*	Upstream
BTA19	30,304,818	30,322,098	ENSBTAG00000003958	*TMEM220*	Upstream
BTA19	30,400,967	30,401,380	ENSBTAG00000046290	*PIRT*	Upstream
BTA19	30,963,518	31,248,936	ENSBTAG00000022509	*DNAH9*	Downstream
BTA19	31,251,615	31,262,941	ENSBTAG00000004675	*ZNF18*	Downstream
BTA19	31,284,737	31,363,093	ENSBTAG00000012103	*MAP2K4*	Downstream
BTA19	31,333,512	31,333,611	ENSBTAG00000030109	*bta-mir-744*	Downstream
BTA28	42,060,434	42,096,557	ENSBTAG00000019194	*FAM35A*	Upstream
BTA28	42,111,714	42,119,577	ENSBTAG00000018356	*SYT15*	Upstream
BTA28	42,138,338	42,139,699	ENSBTAG00000046597	*GPRIN2*	Upstream
BTA28	42,248,870	42,250,003	ENSBTAG00000004760	*PPYR1*	Upstream
BTA28	42,288,945	42,305,211	ENSBTAG00000018499	*ANXA8L1*	Upstream
BTA28	42,317,524	42,364,278	ENSBTAG00000023023	*ANTXRL*	Upstream
BTA28	42,584,644	42,585,670	ENSBTAG00000039154	Novel gene	Upstream
BTA28	42,594,373	42,603,915	ENSBTAG00000005003	*RBP3*	Upstream
BTA28	42,612,012	42,616,128	ENSBTAG00000005004	*GDF2*	Upstream
BTA28	42,618,238	42,618,617	ENSBTAG00000004437	Novel gene	Upstream
BTA28	42,628,171	42,640,763	ENSBTAG00000001019	*GDF10*	Upstream
BTA28	42,862,876	42,895,314	ENSBTAG00000014938	*PTPN20*	Upstream
BTA28	42,939,762	43,003,209	ENSBTAG00000005568	*FRMPD2*	Upstream
BTA28	43,160,797	43,196,195	ENSBTAG00000007876	*MAPK8*	Within peak
BTA28	43,204,020	43,239,165	ENSBTAG00000007878	*ARHGAP22*	Within peak
BTA28	43,426,276	43,657,765	ENSBTAG00000023028	*WDFY4*	Within peak
BTA28	43,603,727	43,620,831	ENSBTAG00000005996	*LRRC18*	Within peak
BTA28	43,677,656	43,749,033	ENSBTAG00000006288	*VSTM4*	Within peak
BTA28	43,774,962	43,778,784	ENSBTAG00000047402	*FAM170B*	Within peak

Chromosome	Start position	End position	Bovine Ensembl ID	Gene symbol	Position relative to selection peak
BTA28	43,803,996	43,807,271	ENSBTAG00000006042	*C10orf128*	Downstream
BTA28	43,930,428	43,934,735	ENSBTAG00000047155	*C10orf71*	Downstream
BTA28	43,961,640	43,993,582	ENSBTAG00000011991	*DRGX*	Downstream
BTA28	44,015,477	44,086,510	ENSBTAG00000032527	*ERCC6*	Downstream
BTA28	44,101,815	44,101,921	ENSBTAG00000042139	*U6*	Downstream
BTA28	44,123,874	44,125,448	ENSBTAG00000014990	*SLC18A3*	Downstream
BTA28	44,143,245	44,187,239	ENSBTAG00000016814	*CHAT*	Downstream
BTA28	44,195,943	44,205,538	ENSBTAG00000045949	Novel gene	Downstream
BTA28	44,224,198	44,251,162	ENSBTAG00000021728	*OGDHL*	Downstream
BTA28	44,283,041	44,397,693	ENSBTAG00000023018	*PARG*	Downstream
BTA28	44,397,814	44,418,582	ENSBTAG00000011694	*TIMM23*	Downstream
BTA28	44,410,273	44,410,472	ENSBTAG00000043162	*SNORA74*	Downstream
BTA28	44,419,336	44,441,664	ENSBTAG00000019565	*NCOA4*	Downstream
BTA28	44,443,910	44,454,333	ENSBTAG00000011660	*MSMB*	Downstream
BTA28	44,495,476	44,542,213	ENSBTAG00000018915	*FAM21A*	Downstream
BTA28	44,545,580	44,597,645	ENSBTAG00000006719	*ZFAND4*	Downstream
BTA28	44,612,704	44,722,610	ENSBTAG00000019537	*MARCH8*	Downstream
BTA28	44,729,723	44,776,997	ENSBTAG00000020319	*ALOX5*	Downstream

Supplementary Table 6: Enriched subcategories in the IPA *Physiological System Development and Function* category for genes located ± 1 Mb of selection scan peaks (Excel file - **Supp_table_06.xlsx**).
